# Supplementary material for: Gender and racial diversity socialization in science
Source: Nat Comput Sci. 2025 Apr 17;5(6):481–91. doi: 10.1038/s43588-025-00795-9 (PMC12187764; doi:10.1038/s43588-025-00795-9)
Supplement: Supplementary file 1 — Supplementary Figs. 1–26, Discussion and Notes, and Tables 1–8. [file 43588_2025_795_MOESM1_ESM.pdf]

nature computational science

Supplementary information

https://doi.org/10.1038/s43588-025-00795-9

Gender and racial diversity socialization in science

In the format provided by the authors and unedited

Contents

Supplementary Note 1 Data description **2**

Supplementary Note 2 Gender diversity socialization in mentorship **3**

Supplementary Note 2.1 Descriptive analyses 3

Supplementary Note 2.2 Randomized null model for mentorship data 5

Supplementary Note 2.3 Gender diversity in mentorship under the null model 7

Supplementary Note 2.4 Regression analyses 9

Supplementary Note 3 Gender diversity socialization in coauthorship networks **16**

Supplementary Note 3.1 Gender and author names 16

Supplementary Note 3.2 Descriptive analyses 18

Supplementary Note 3.3 Randomized null model for coauthorship data 19

Supplementary Note 3.4 Gender diversity in coauthorship under the null model 22

Supplementary Note 3.5 Other robustness tests 25

Supplementary Note 3.6 Regression analyses 28

Supplementary Note 4 Racial diversity socialization **35**

Supplementary Note 4.1 Racial diversity socialization in coauthorship under the null model 35

Supplementary Note 4.2 Other robustness tests 37

Supplementary Note 4.3 Regression analyses 38

Supplementary Note 5 Sociological theory and implications **46**

Supplementary Note 6 Intersectional analysis of gender and race **50**

Supplementary Note 1 Data description

We assess the diversity association effect in science using two complementary large-scale data sets, one containing a set of known advisor-advisee relationships and one for which we infer such relationships from coauthorship patterns in a large bibliographic database. The first data set is comprised of start and end years for 339,744 advisor-advisee pairs spanning STEM fields, social sciences, and humanities[1]. Within these data, we analyze the career trajectories of 17,917 individual researchers for whom we can track and identify at least one of their advisors and at least one advisee they subsequently advised as established researchers. The majority of these researchers were affiliated with North American or European institutions and started their training period from 1970 to 2010. Using a standard name-based algorithm, we assign a binary gender label to 4,070 women and 13,847 men, where men compose a disproportionate 77.3% of all these researchers (see Methods). A binary gender classification is used to facilitate large-scale statistical analysis and does not provide a full accounting of gender identities, which would require self-identifications[2,3]. Individuals with gender-ambiguous names are omitted from the study.

The second data set is derived from records of 30.6 million research articles in the Microsoft Academic Graph (MAG) database from 1950 to 2021, covering publications in natural sciences, engineering, mathematics, and social sciences[4]. We first construct the coauthorship networks of researchers with at least 10 years between the first and most recent publication and at least 10 publications total. To these, we apply the same name-based gender labeling algorithm to assign binary gender labels to the 562,494 authors that have junior collaborators in both the early career and established period (32.3% women and 67.7% men). Additionally, for this data set, we apply a name-based race/ethnicity labeling algorithm to assign racial group labels to authors. We note that such algorithms have uneven accuracy in ascribing race to scholars compared to self-identified demographics[5], due to the historically contingent nature of current racial categories and their poor

alignment with the diverse cultural associations of names. Thus, we use predicted probabilities in aggregate racial estimates of researchers rather than discrete classifications. This process results in 855,526 researchers who have collaborated with at least one junior coauthor in both the early career and established period. The selected author set encompasses researchers from worldwide institutions, with the majority based in North America, Europe, and Asia. Our approach to approximate racial diversity at the aggregate level cannot be applied to small research groups of only a few members (see Methods), and hence we do not apply it to the mentorship data in which this pattern is very common.

We augment these two data sets by assigning prestige levels to institutions based on their historical publication records. Within a specific subfield, institutional prestige is ranked by the total number of highly-cited articles, which is defined as papers receiving the upper 5th percentile of citations two years after publication. We then divide institutions into 4 groups, the top tier being the upper 1%, the second tier being the upper 2% to 10%, the third tier being the upper 11% to 20%, and the remainder being the fourth tier.

Supplementary Note 2 Gender diversity socialization in mentorship

Supplementary Note 2.1 Descriptive analyses

We provide descriptive statistics for the effects of gender diversity association on mentorship data. We show the number of established researchers selected for the analysis over time for men and women based on the proportion of women advisees in their advisors' research group, as a function of their first year in the training period (Supplementary Figure 1**a**). Because the selected

researchers must have appeared in the data as both the advisee and the advisor, the number of researchers in the data set peaks around 2000 and sharply declines thereafter. The largest group is men researchers with fewer than 50% women advisees in the advisor's group, while women researchers are often trained with more than 50% women advisees in the advisor's group. Regarding advisor group size, we find that most advisees were trained with a limited number of other advisees, yet there are a few exceptional researchers who received training in very large groups with over 30 advisees (Supplementary Figure 1**b**).

To further explore the relation between advisor group size and the gender diversity association effects, we break down the cohort of selected established researchers into 4 groups. We find that the gender diversity association effect is larger for men researchers trained in groups with 4 or more advisees than those trained in small groups with 1-3 advisees (Supplementary Figure 2). This effect is more prominent when the group size is 4-6 or 7-10, for both men and women researchers. This result suggests that the effect of gender diversity association may be enhanced in relatively large research groups.

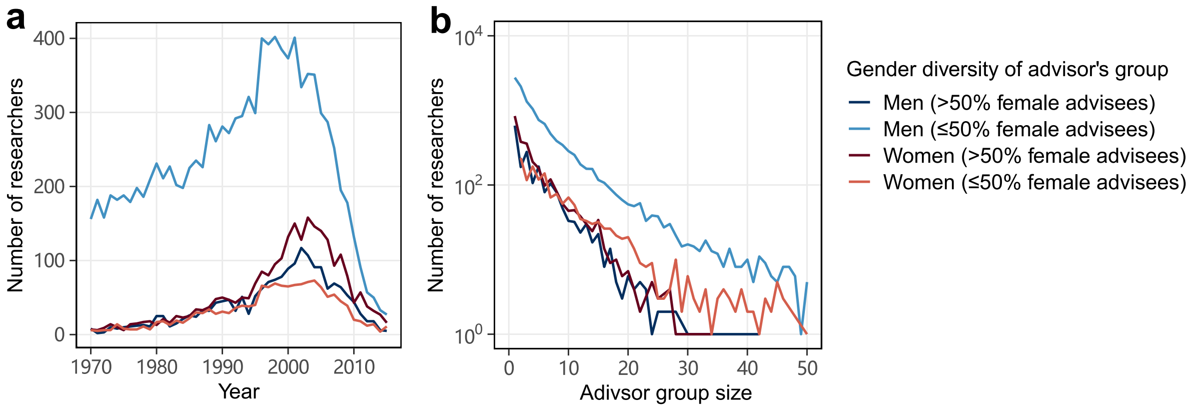

Supplementary Figure 1. **Descriptive statistics of the mentorship data.** **a**, the number of established researchers selected for the analysis over time. **b**, the number of selected researchers as a function of the group size in their training period.

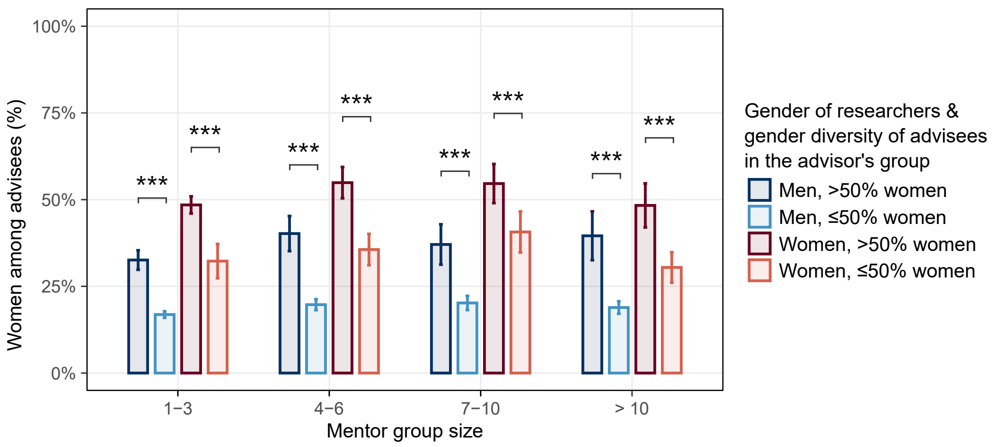

Supplementary Figure 2. **Gender diversity association effect by group size in the mentorship data.** As a descriptive analysis, we use the 50% threshold to determine gender diversity. We divide groups in which the selected researchers were trained into four bins according to the number of advisees ($n = 17,917$). Bars represent mean values and error bars indicate 95% confidence intervals. Two-sided t-tests are used for multiple comparisons. ($^{***}p < 0.001$; $^{**}p < 0.01$; $^{*}p < 0.05$).

Supplementary Note 2.2 Randomized null model for mentorship data

We propose a new randomized null model to examine the expected proportion of women in a research group. As we discuss in the main text, the demographic composition of the scientific workforce can vary, and it is dependent on a number of exogenous factors such as time, subfield, country, and institutional prestige. The demographic composition of the scientific workforce varies substantively over time and across disciplines, institutions, and geographical boundaries. For instance, the proportion of women in science has gradually increased over the past 40 years[6], but has done so unevenly across subfields and institutions[7], with more women in biology and chemistry and fewer in mathematics, physics, and computer science[8]. An engineering department with 10% women faculty in 1950 could be quite gender-diverse, while in 2020, most of these departments hire more than 15% of women among their faculty[7]. Field of study can be another critical factor in determining the level of expected gender diversity. A medical research group with 40% of women

is common, as medicine is a fairly gender-balanced field. In contrast, a research group in computer science with 30% women is quite gender diverse, as computer science is heavily dominated by men researchers.

Hence, we should expect established researchers to have more gender (or racially) diverse junior collaborators than their own training environment simply because of this overall increase in women's representation (and racial diversity), even if there is no diversity association effect. Therefore, we introduce a null model to estimate an appropriate baseline for the expected diversity among junior collaborators due only to the random mixing of established and junior researchers, conditioned on the available demographic diversity in the population at that time.

There are a few potential reasons why some advisors choose to train a relatively large proportion of women among their advisees. It could be that this advisor has a personal preference to nurture a gender-diverse environment in the research group. It could also be that having many women advisees is simply because more women are available under particular structural characteristics of the environment. For example, some fields, such as medicine and biology, historically attract more women students than mathematics. As more women attend graduate training programs over time, there are more women advisees in the higher education system than what it was two decades ago. Other factors, such as country of origin and institutional prestige, may also come into play when determining the gendered composition of the scientific workforce. Thus, implementing a randomized null model under constraints controlling for these confounding factors is necessary to reflect an expected gender diversity in specific research environments.

In the mentorship data, we have the yearly pairings of advisor-advisee in natural sciences, arts & humanities, and social sciences over 4 decades. An advisee receives training in an advisor's group for a few years (see Fig. Supplementary Figure 3). To randomize the mentorship network, we first select an advisor-advisee pair (i_1, i_2), and randomly choose another advisor-advisee pair

(j_1, j_2) which was also active in the same year (see Algorithm 1). Then we check other conditions, including whether advisees i_2 and j_2 have at least one common subfield, whether they come from the same country, and whether their institutions are at the same tier of institutional prestige. If all these conditions are met, we then reshuffle these advisor-advisee pairs, and the new pairing becomes (i_1, j_2) and (j_1, i_2). We repeat this process for all advisor-advisee pairs for each iteration of the network null model randomization. We run 100 iterations to make the new advisor-advisee pairing adequately randomized under the constraints. Then we take one snapshot of the mentorship network under the null model and calculate the gender diversity of advisees. We repeat this procedure and take 100 snapshots of the mentorship network under the null model.

For a given researcher i, we define ρ_i as the observed proportion of women in i's group and $\langle \rho_i^{\text{null}} \rangle$ is the average proportion of women expected under the 100 snapshots of the null model. If researcher i satisfies

$$\rho_i > \langle \rho_i^{\text{null}} \rangle, \tag{1}$$

then we say i has a high percentage of women advisees compared to the null model.

Supplementary Note 2.3 Gender diversity in mentorship under the null model

We show the proportion of researchers that had a high percentage of women in the advisors' groups relative to the null model over time, and this trend steadily increases from the 1970s to 2010s (Supplementary Figure 4**a**). Then we show the proportion of advisor groups with a high percentage of women by group size, indicating that this trend is relatively stable as a function of research group size. (Supplementary Figure 4**b**).

To further study how the advisor group size influences the gender diversity association effects, we classify all established researchers into 4 groups by the advisor group size. Under the null

Algorithm 1 Randomized null model for mentorship data

Input: Mentorship network M, model number of replication R
Output: Randomized mentorship network $\hat{M}$
Initialize randomized network $\hat{M}$ as M
while total replications is smaller than R **do**
  for each advisee i from $\hat{M}$ in year y **do**
    Randomly select another advisee j from $\hat{M}$ in year y
    Mark p as normal
    if i and j have no common subfield **then**
      Mark p as anomaly
      continue ▷ Skip to the next advisee
    else if i and j from different countries **then**
      Mark p as anomaly
      continue
    else if i and j's institutions not within the same institutional prestige tier **then**
      Mark p as anomaly
      continue
    end if
    if p is normal **then**
      Reshuffle position of advisees i and j in year y in $\hat{M}$ ▷ Exchange their advisors
    end if
  end for
end while

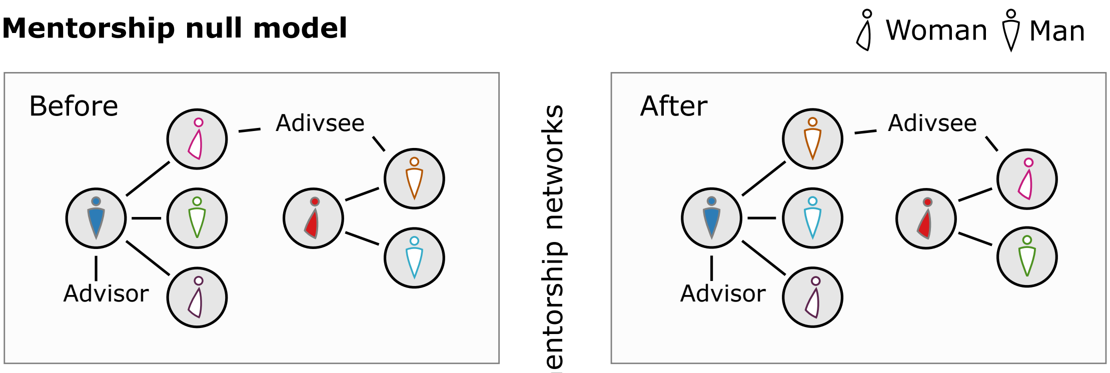

Advisor	Advisee	Year
B. E. Simons	A. J. Miles	1998
B. E. Simons	C. K. Owen	2003
B. E. Simons	T. Jones	2006
A. Johnson	J. R. Smith	1998
A. Johnson	C. Anthony	2003

Advisor	Advisee	Year
B. E. Simons	J. R. Smith	1998
B. E. Simons	C. Anthony	2003
B. E. Simons	T. Jones	2006
A. Johnson	A. J. Miles	1998
A. Johnson	C. K. Owen	2003

Supplementary Figure 3. **Illustration of the randomized null model for mentorship networks.** We first construct the original advisor-advisee network, and then reshuffle advisees, controlling for a range of factors that may influence the demographic composition of researchers, including time, subfield, country, and institutional prestige.

model setting, the group of researcher i has high percentage of women advisees when $\rho_i > \langle\rho_i^{\text{null}}\rangle$. The gender diversity association effect is larger for men researchers trained in large groups (Supplementary Figure 5). This effect is more obvious when the group size is 4-6 or 7-10, for both men and women researchers. This result suggests that the effect of gender diversity association under the null model setting is more substantial in relatively large research groups.

Supplementary Note 2.4 Regression analyses

To further validate the observed gender diversity association effects in both descriptive analyses and the analyses using the null model as an expected value, we conduct several regression analyses

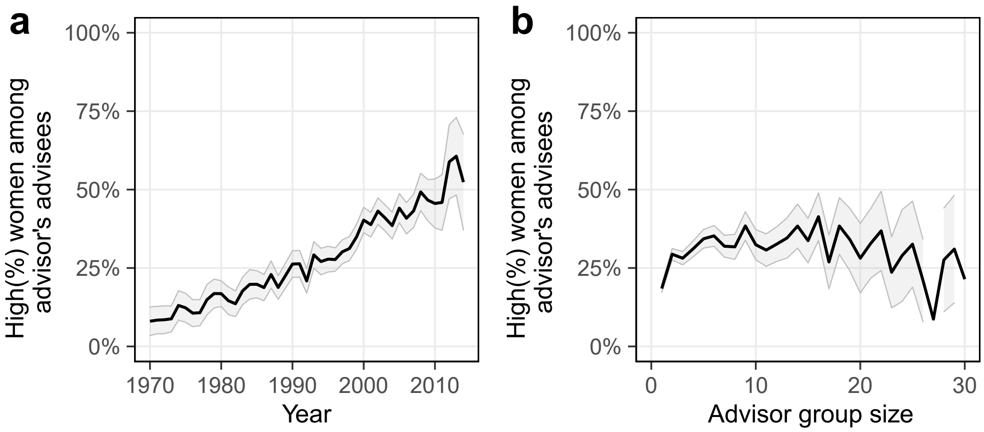

Supplementary Figure 4. **High percentage of women in the advisors' groups relative to the null model.** **a**, the proportion of advisor groups that have a high percentage of women relative to the null model over time. **b**, the proportion of advisor groups with a high percentage of women as a function of group size. Lines denote mean values and shaded areas represent 95% confidence intervals.

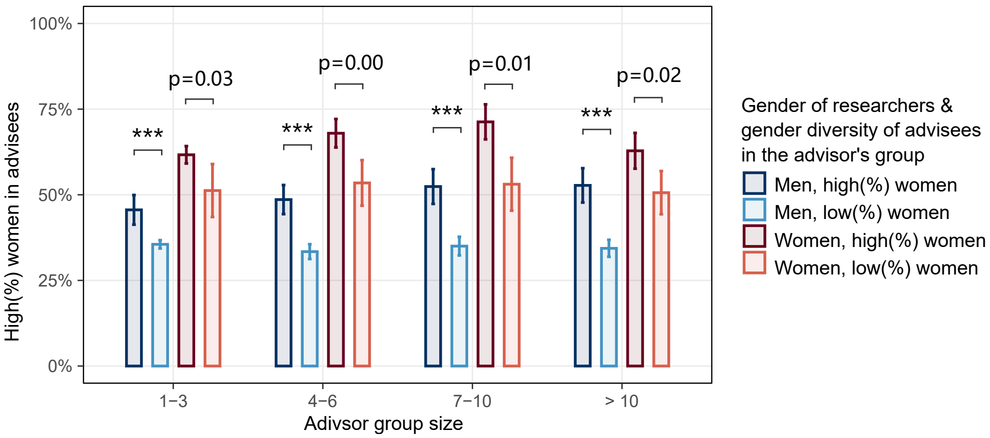

Supplementary Figure 5. **Gender diversity association effect in the mentorship data relative to the null model as a function of group size.** We divide research groups in which the selected researchers were trained into four bins according to the number of advisees ($n = 17,917$). Bars represent mean values and error bars indicate 95% confidence intervals. Two-sided t-tests are used for multiple comparisons. ($^{***}p < 0.001$; $^{**}p < 0.01$; $^{*}p < 0.05$).

controlling for a series of factors that may affect the gender diversity of advisees the researchers train in the established period. Gender diversity of advisees may be related to the culture and norms

of specific research institutions, as studies have uncovered facts of how prestige shapes gendered performance and career prospects in science[9,10]. Therefore, we include institutional prestige in the regression analyses. In this paper, we divide institutions into 4 groups: the top tier being the upper 1%, the second tier being the upper 2% to 10%, the third tier being the upper 11% to 20%, and the remainder being the fourth tier[11]. This is done by assigning prestige levels to institutions based on their historical publication records within a subfield. Specifically, institutions are ranked by the number of highly-cited articles they have published, defined as papers receiving the upper 5th percentile of citations two years after publication.

Gender diversity association effects could be related to the advisor group size, which is a key factor that affects team performance in science[12,13]. For instance, large gender-diverse research teams may provide more interaction opportunities between men and women advisees. As the size of teams increases, it may also shift the underlying working mechanisms of teams, the type of research questions they probe, and the novelty of research these teams produce[14]. Therefore, we include this variable as a factor that may influence the propensity of researchers to cultivate gender-diverse research groups in the established period. Homophily is a key factor in forming social networks, and women researchers are more likely to recruit more women advisees when they establish research groups. In the analyses, we include a binary variable to indicate whether the focal researcher is a woman or not.

We include two other variables of gender diversity at the subfield and country levels, as the workforce composition can alter substantially across academic disciplines and geographic borders. For instance, some fields like biology are more gender-balanced than fields such as physics and engineering[15,16]. As the likelihood of recruiting women advisees is closely related to the proportion of women students within the specific subfield, we include the proportion of women advisees by subfield as a control variable. Similarly, the involvement of women in science divergences

across countries and geographic regions[17], and the chance to train women advisees is affected by the proportion of women students in a particular country. We include the proportion of women advisees by country as a control variable.

The key variable to analyze is how the gender diversity in the advisor group during the training period affects the level of women advisees trained by researchers in the established period. We first use linear regression models to examine how the women percentage in the advisor's group during the training period of researchers predicts the outcome variable, which is the women percentage of advisees when they become established researchers (Supplementary Table 1). Several control variables, including researcher gender, the proportion of women advisees in the subfield, and the proportion of women advisees by country, all have strong positive relations with the women percentage among researchers' advisees. Regarding the key variable of women percentage in the training period, we find that women percentage in the advisor's group during their training period has a significantly positive relation with the outcome variable, which is consistent across all linear regression models (Supplementary Table 1 models 2-4).

Under the null model setting, we then use logistic regression models to examine how having a high percentage of women in the advisor's group during the training period of researchers predicts the outcome variable, which is a binary coding of whether the women percentage of advisees is high relative to the null model when they become established researchers (Supplementary Table 2). Similarly, several control variables including researcher gender, the proportion of women advisees in the subfield, and the proportion of women advisees by country all have strong positive relations with having high women percentage among researchers' advisees (Supplementary Table 2 model 8). For instance, when the focal researcher is woman, she has an odds ratio of 1.759, suggesting that she has on average 75.9% more likely to train a high percentage of women advisees compared to the null model. More importantly, we find that having high percentage of

women in the advisor's group during their training period has significantly positive relation with the outcome variable. The odds ratio of being trained in high percentage of women group is 1.369, suggesting that researchers whose advisor group has high percentage of women are 36.9% more likely to cultivate gender diverse research environments when they become established researchers. The diversity socialization factor in the training period is consistently significant across all logistic regression models (Supplementary Table 2 models 6-8).

These results suggest that, using either the crude proportion of women advisees in the advisor's group or the binary coding of whether the researcher's advisor trained a high percentage of women advisees relative to the null model, can validate the gender diversity association effect of how the gender diversity experience in the early training period affects the diversity preferences of the researchers in the established period. The effects remain significant when we control for several potentially confounding factors, including time, the size of the advisor's group, and other gender-related features such as the gender of the focal researcher, and the gender composition of advisees within a subfield and country.

Supplementary Table 1. **Linear regression models to predict the proportion of women among researchers' advisees.** Two-sided t-tests are used for multiple comparisons.

Dependent variable:	Women (%) among researchers' advisees			
Model:	(1)	(2)	(3)	(4)
(Intercept)	0.329^{***}	0.304^{***}	0.039^{*}	-0.181^{***}
	(0.018)	(0.018)	(0.019)	(0.031)
Institutional prestige	-0.018	-0.019	-0.038^{*}	-0.037^{*}
	(0.019)	(0.019)	(0.018)	(0.018)
Advisor group size	0.000	-0.000	0.001	0.001
	(0.000)	(0.000)	(0.000)	(0.000)
Researcher is woman	0.194^{***}	0.087^{***}	0.084^{***}	0.083^{***}
	(0.007)	(0.009)	(0.009)	(0.009)
Women(%) by subfield			0.845^{***}	0.830^{***}
			(0.024)	(0.024)
Women(%) by country				0.637^{***}
				(0.070)
Women(%) in advisor's group		0.216^{***}	0.122^{***}	0.118^{***}
		(0.012)	(0.012)	(0.012)
R^2	0.053	0.071	0.140	0.145
Adj. R^2	0.053	0.071	0.140	0.144
Num. obs.	15637	15637	15637	15637

Robust standard-errors in parentheses

Signif. Codes: $^{***}p < 0.001$; $^{**}p < 0.01$; $^{*}p < 0.05$

Supplementary Table 2. **Logistic regression models to predict whether researchers have high (%) of women advisees compared to the null model.** Two-sided t-tests are used for multiple comparisons.

Dependent variable:	High (%) women advisees in established period			
Model:	(5)	(6)	(7)	(8)
(Intercept)	-0.419^{***}	-0.507^{***}	-2.168^{***}	-3.835^{***}
	(0.112)	(0.112)	(0.130)	(0.226)
Institutional prestige	-0.114	-0.115	-0.237	-0.225
	(0.116)	(0.116)	(0.121)	(0.121)
Advisor group size	-0.000	0.000	0.005^{*}	0.004^{*}
	(0.002)	(0.002)	(0.002)	(0.002)
Researcher is woman	0.990^{***}	0.647^{***}	0.574^{***}	0.565^{***}
	(0.040)	(0.048)	(0.050)	(0.050)
Women(%) by subfield			5.107^{***}	5.002^{***}
			(0.170)	(0.171)
Women(%) by country				4.779^{***}
				(0.525)
High(%) women in advisor's group		0.570^{***}	0.319^{***}	0.314^{***}
		(0.044)	(0.046)	(0.046)
AIC	20681.724	20514.046	19514.424	19426.272
BIC	20712.353	20552.333	19560.369	19479.874
Log Likelihood	-10336.862	-10252.023	-9751.212	-9706.136
Deviance	20673.724	20504.046	19502.424	19412.272
Num. obs.	15637	15637	15637	15637

Robust standard-errors in parentheses

Signif. Codes: $^{***}p < 0.001$; $^{**}p < 0.01$; $^{*}p < 0.05$

Supplementary Note 3 Gender diversity socialization in coauthorship networks

Supplementary Note 3.1 Gender and author names

We adopt a name-based gender assignment method to infer gender of researchers. We use the first names of authors from the publication data and match them with the gender association obtained from demographic census data[18]. This method does not apply to authors that use only initials of first names, which we show in Supplementary Figure 6**a**. Physicists have the highest preference of initial letter usage, and only about half of them write full names in their work. In natural science and engineering fields, about one-fourth of researchers use name initials, and social scientists generally have the lowest ratio of name initial usage. Fields with less frequent use of name initials have a higher proportion of authors eligible for the name-based gender assignment procedure.

Among the researchers that we assign gender, we show the proportion of women by field in Supplementary Figure 6**b**. Researchers in humanities and social science fields have the highest women ratio, especially in arts, psychology, and sociology. Some natural science fields, such as biology and medicine, also have a more gender-balanced workforce, with about half of researchers being women. Computer science, engineering, mathematics, and physics are among the fields with the lowest women percentage, with only one out of four researchers being women.

The proportion of women researchers has been increasing over the past five decades (Supplementary Figure 6**c**). However, the speed of growth over time is heterogeneous across fields, with some arts & humanities and social science fields having higher growth rates, while some engineering fields such as computer science have more moderate growth rates of women researchers. Some fields, such as biology, have reached a gender balance and have remained stable over the

past decade or two.

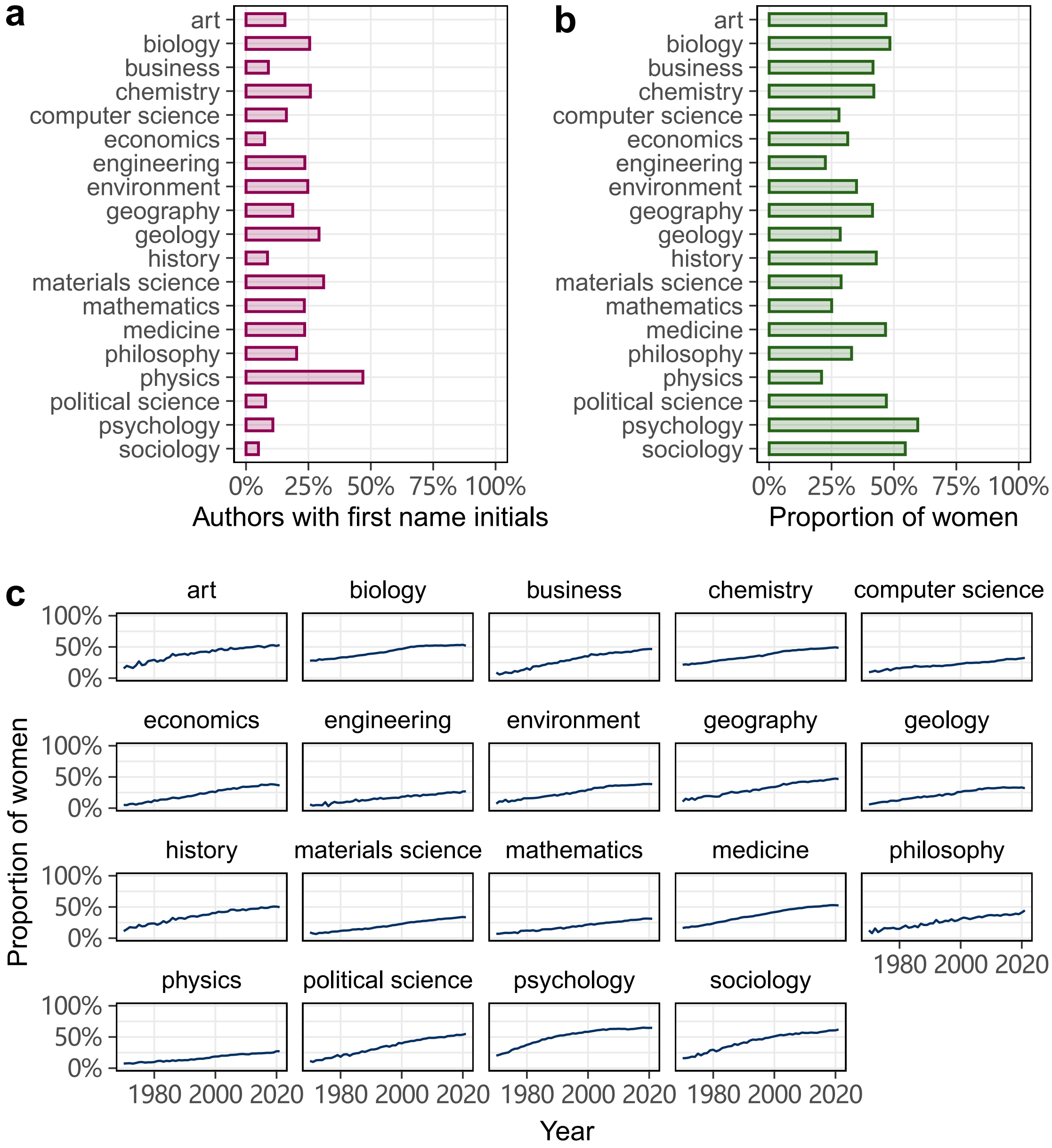

Supplementary Figure 6. **Descriptive statistics of the author names and gender composition.** **a**, proportion of authors with name initials in the publication data. **b**, proportion of women among authors that are assigned gender using their first names. **c**, time dynamics of the proportion of women among researchers based on the first year of publication.

Supplementary Note 3.2 Descriptive analyses

We present descriptive statistics of the selected established researchers in the coauthorship networks. Using a naive 50% threshold to determine the gender diversity of coauthors in this descriptive analysis, we divide researchers into 4 groups based on researcher gender and gender diversity of early coauthors. We show the number of established researchers in each group by the first publishing year over time (Supplementary Figure 7**a**). While the number of researchers in all groups increase over time, for both men and women, they are more likely to work with men junior coauthors in the early-career. However, by 2010, women researchers are almost equally likely to work with men and women junior coauthors. We show the number of established researchers as a function of the number of their junior coauthors in the early-career (Supplementary Figure 7**b**). While most researchers have fewer than 10 early coauthors, a few exceptional researchers worked with more than 50 junior coauthors in the early career.

We show the gender diversity association effect in STEM, arts & humanities, and social sciences, and find that the gender diversity association effects varies substantially across fields (Supplementary Figure 8). For instance, in some fields where there is a higher proportion of women researchers, such as biology, medicine, and social sciences, both men and women are more likely to have more than 50% of coauthors being women. Whereas in fields like computer science, engineering, and physics that traditionally attract fewer women, both men and women researchers are less likely to collaborate with women. Nevertheless, except for history, we find that the diversity association effect persists in all other fields.

To further explore the relation between the number of early coauthors and the gender diversity association effects, we break down the cohort of selected established researchers into 4 groups by coauthor number. We find that the gender diversity association effect is larger for men

researchers who worked with more junior coauthors in the early career (Supplementary Figure 9). This effect is more significant when the number of early coauthors is more than 20, for both men and women researchers. This result suggests that the gender diversity association effect may be more pronounced when researchers collaborated with a large number of coauthors in the early career.

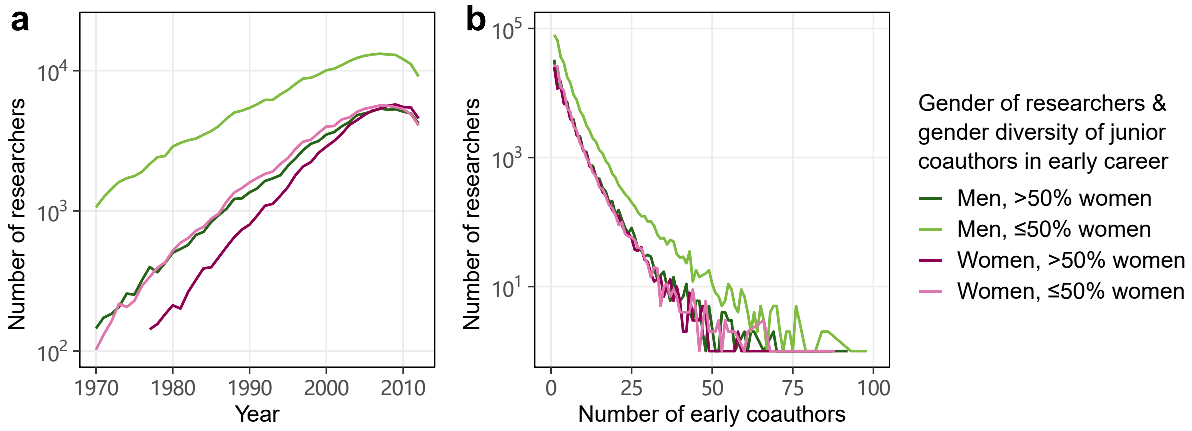

Supplementary Figure 7. **Descriptive statistics for the coauthorship network.** **a**, we show the number of established researchers selected for the analysis over time. **b**, we show the number of established researchers as a function of the number of their junior coauthors in the early career. We use the 50% threshold to determine the gender diversity of coauthors in this descriptive analysis.

Supplementary Note 3.3 Randomized null model for coauthorship data

We propose a new randomized null model to examine the expected proportion of women among a focal researcher's coauthors. A researcher collaborating with a relatively large proportion of women coauthors may be due to a few reasons. It could be that this researcher has a high propensity to work with a gender-diverse cohort of coauthors. It could also be that having many women coauthors is simply because more women are available in these particular structural properties of the environment. For example, some fields such as medicine naturally have more women than fields like computer science, thus it is more likely to work with women in medicine than in com-

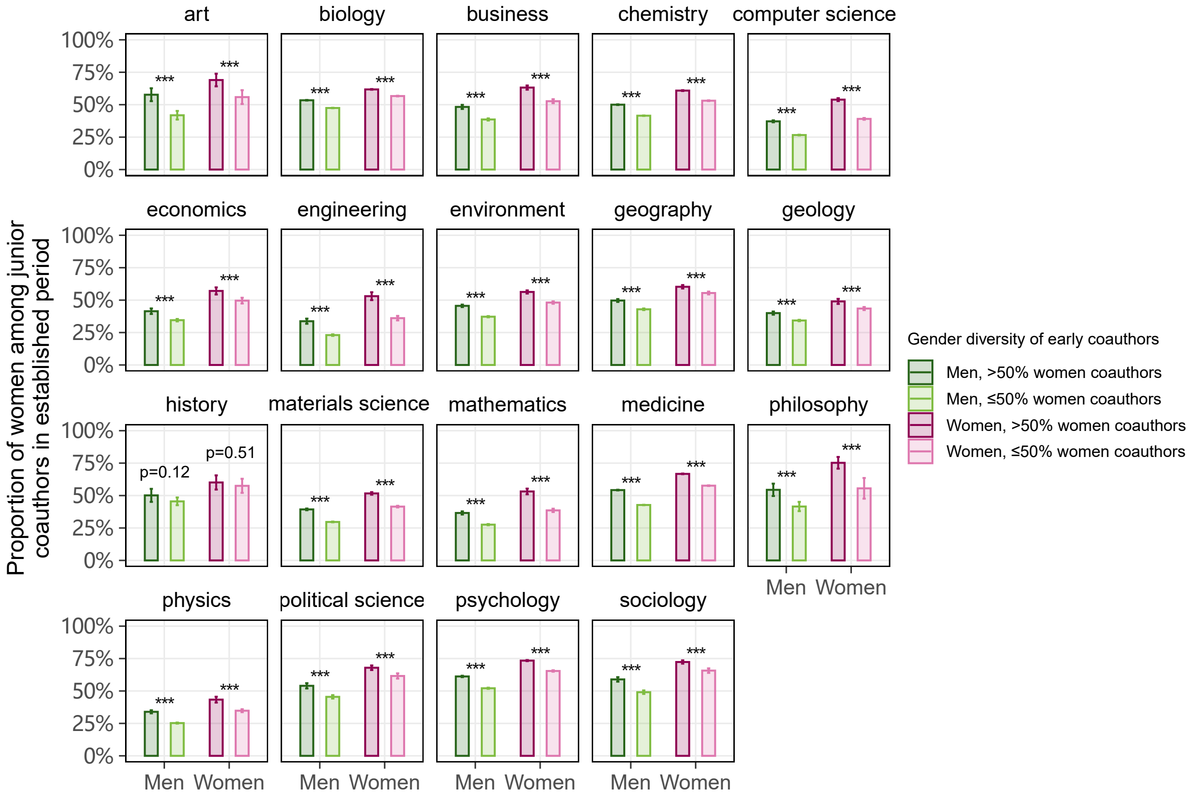

Supplementary Figure 8. **Descriptive statistics of the diversity association effects.** We use 50% of women as a naive threshold for gender diversity in the early career, and show how the diversity association effect varies by research fields in STEM, arts & humanities, and social sciences ($n = 562,494$). Bars represent mean values and error bars indicate 95% confidence intervals. Two-sided t-tests are used for multiple comparisons. (***$p < 0.001$; **$p < 0.01$; *$p < 0.05$; NS, not significant).

puter science. As more women attend graduate training programs over time, there are more women junior researchers than two decades ago. Other factors, such as country of origin and institutional prestige, may also come into play when determining the gendered composition of the scientific workforce. Thus, implementing a randomized null model under constraints controlling for these confounding factors is necessary to reflect the level of gender diversity expected in a specific research environment.

The construction of the coauthorship network is slightly different from the mentorship data,

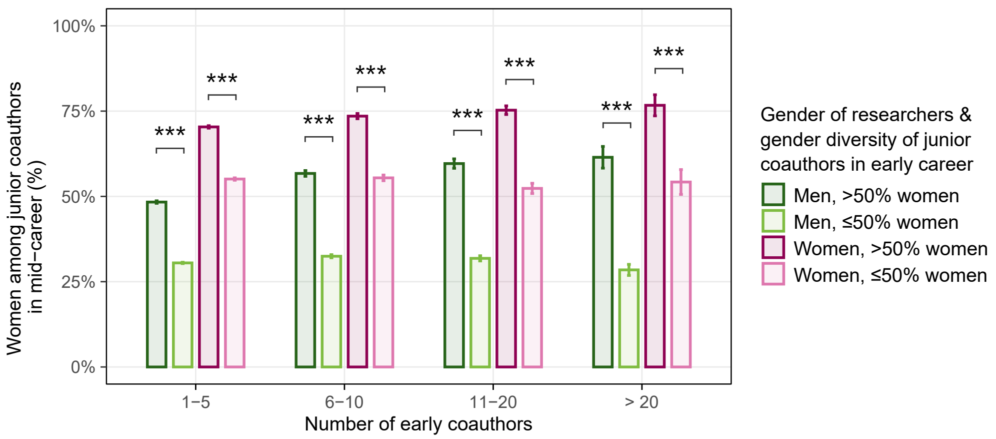

Supplementary Figure 9. **Gender diversity association effect in the coauthorship network as a function of the number of early coauthors.** We use the 50% threshold to determine the gender diversity of coauthors in this descriptive analysis ($n = 562,494$). Bars represent mean values and error bars indicate 95% confidence intervals. Two-sided t-tests are used for multiple comparisons. ($^{***}p < 0.001$; $^{**}p < 0.01$; $^{*}p < 0.05$).

which we illustrate in Supplementary Figure 10. To randomize the coauthorship network, we first select a coauthor pair (i_1, i_2), and randomly choose another coauthor pair (j_1, j_2) which was also active in the same year (see Algorithm 2). Then we check other conditions, including whether researchers i_2 and j_2 have at least one common subfiled, whether they come from the same country, and whether their institutions are at the same tier of institutional prestige. If all these conditions are met, we then reshuffle these coauthor pairs, and the new pairing becomes (i_1, j_2) and (j_1, i_2). We repeat this process for all coauthor pairs for each iteration of the network null model randomization. We run 100 iterations to make the new coauthor pairing adequately randomized under the constraints. Then we take one snapshot of the coauthorship network under the null model and calculate the gender diversity of coauthors. We repeat this procedure and take 100 snapshots of the coauthorship network under the null model.

For a given researcher i, we define ρ_i as the observed proportion of women among i's coauthors and $\langle \rho_i^{\text{null}} \rangle$ is the average proportion of women expected under the 100 snapshots of the null

model. If researcher i satisfies $\rho_i > \langle \rho_i^{\text{null}} \rangle$ then we say i has a high percentage of women coauthors compared to the null model.

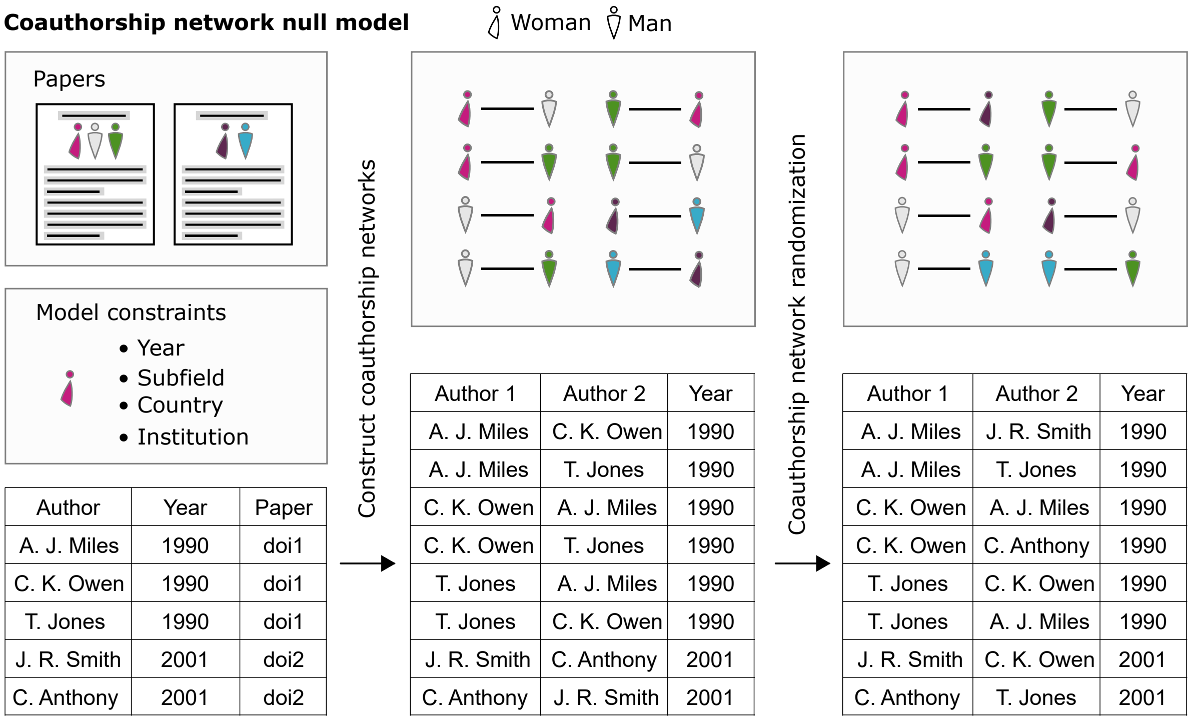

Supplementary Figure 10. **Illustration of the randomized null model for coauthorship networks.** In this example, we pick up two papers that have a total number of five researchers. We first construct the original coauthorship network, as shown in the middle panel. To randomize the coauthorship network, we reshuffle the author order on the right side of the network, and obtain a randomized configuration in the right panel.

Supplementary Note 3.4 Gender diversity in coauthorship under the null model

We show the proportion of researchers that had a high percentage of women among their junior coauthors in the early career relative to the null model by the first publishing year (Supplementary Figure 11**a**). This trend has steadily increased since the 1970s and approached 50% parity in the 2010s. Then we show the proportion of researchers with a high percentage of women early

Algorithm 2 Randomized null model for coauthorship data

Input: Coauthorship network C, model number of replication R
Output: Randomized mentorship network $\hat{C}$
Initialize randomized network $\hat{C}$ as C
while total replications is smaller than R **do**
  for each coauthor pair (i_1, i_2) from $\hat{C}$ in year y **do**
    Randomly select another coauthor pair (j_1, j_2) from $\hat{C}$ in year y
    Mark p as normal
    if i_1 and i_2's paper has no common subfield with j_1 and j_2's paper **then**
      Mark p as anomaly
      continue ▷ Skip to the next coauthor pair
    else if i_2 and j_2 come from different countries **then**
      Mark p as anomaly
      continue
    else if i_2 and j_2's institutions not within the same institutional prestige tier **then**
      Mark p as anomaly
      continue
    end if
    if p is normal **then**
      Reshuffle position of coauthors i_2 and j_2 in year y in $\hat{C}$ ▷ Exchange coauthor pair to (i_1, j_2) and (j_1, i_2)
    end if
  end for
end while

coauthors by the number of coauthors, indicating that this trend is relatively stable as a function of the number of early coauthors (Supplementary Figure 11**b**).

We use $\Delta^{\text{gender}}_{\text{early}} = \rho_{\text{early}} - \langle \rho^{\text{null}}_{\text{early}} \rangle$ to denote the difference between the gender diversity of a researcher's early coauthors relative to the null model. We examine the diversity socialization effects $\Delta^{\text{gender}}_{\text{established}}$ in the established period as a function of gender diversity $\Delta^{\text{gender}}_{\text{early}}$ among early career coauthors. We find that women have an average $\Delta^{\text{gender}}_{\text{early}} \simeq 0$, suggesting that women tend to have a cohort of early coauthors close to the expected level of gender diversity (Supplementary Figure 12**a**). In contrast, men's early coauthors have an average $\Delta^{\text{gender}}_{\text{early}} \simeq -0.2$, which is below the expected gender diversity in the environment. While we have demonstrated the existence of gender diversity association effect in the coauthorship network, which is $P(\Delta^{\text{gender}}_{\text{established}} > 0 | \Delta^{\text{gender}}_{\text{early}} > 0) > P(\Delta^{\text{gender}}_{\text{established}} > 0 | \Delta^{\text{gender}}_{\text{early}} \leq 0)$, the association between $\Delta^{\text{gender}}_{\text{established}}$ and $\Delta^{\text{gender}}_{\text{early}}$ is complicated (Supplementary Figure 12**b**). When $-0.2 < \Delta^{\text{gender}}_{\text{early}} < 0.5$, increasing $\Delta^{\text{gender}}_{\text{early}}$ leads to a positive growth of $\Delta^{\text{gender}}_{\text{established}}$. In other ranges, however, $\Delta^{\text{gender}}_{\text{early}}$ and $\Delta^{\text{gender}}_{\text{established}}$ appears to have a negative correlation.

To further study how the number of early coauthors influences the gender diversity association effects, we divide these established researchers into 4 groups by the number of early coauthors. Under the null model setting, we define researcher i to have a high percentage of women early coauthors when $\rho_i > \langle \rho^{\text{null}}_i \rangle$. We find that the gender diversity association effect is larger for men researchers who worked with a large number of early coauthors (Supplementary Figure 13). This effect is more prominent when the number of early coauthors is over 20, for both men and women researchers. This result suggests that the gender diversity association effect under the null model setting is stronger in relatively large research groups.

We show the gender diversity association effect under the null model setting in STEM, arts & humanities, and social sciences, and find that the gender diversity association effects varies sub-

stantially across fields (Supplementary Figure 14). In some fields where there is a higher proportion of women researchers, such as biology, medicine, and social sciences, both men and women are more likely to have worked with a high percentage of women coauthors relative to the null model in the early career. Whereas in fields like computer science, engineering, and physics that traditionally attract fewer women, both men and women researchers are less likely to work with a high percentage of women coauthors. We find that the diversity association effect persists in all fields except for history, and the effect is more prominent for men researchers.

We further examine how the gender diversity association effect varies in four major domains, i.e., arts & humanities, engineering & mathematics, natural sciences, and social sciences (Supplementary Figure 15). The gender diversity association effect is not significant in arts & humanities, which includes arts, history, and philosophy, especially for researchers with a large number of early coauthors. In contrast, natural sciences have substantial diversity association effect for both men and women. This may be due to the group dynamics difference in these fields, where students tend to work on individual research projects in arts & humanities, while there is more collaborative work for students in natural sciences, especially in biology and physics, where large research groups have become more common.

Supplementary Note 3.5 Other robustness tests

We conduct a robustness test by changing the definition of the established period. In the main paper, we define established researchers as those who have at least 6 publishing career years. Here, we modify the definition of the established period to 10 or more publishing years. We find that the gender diversity association effect under the null model setting persists for both men and women established researchers (Supplementary Figure 16**a**). The gender diversity association effect by the first publishing year of researchers over time is relatively stable, and the effect is more remarkable

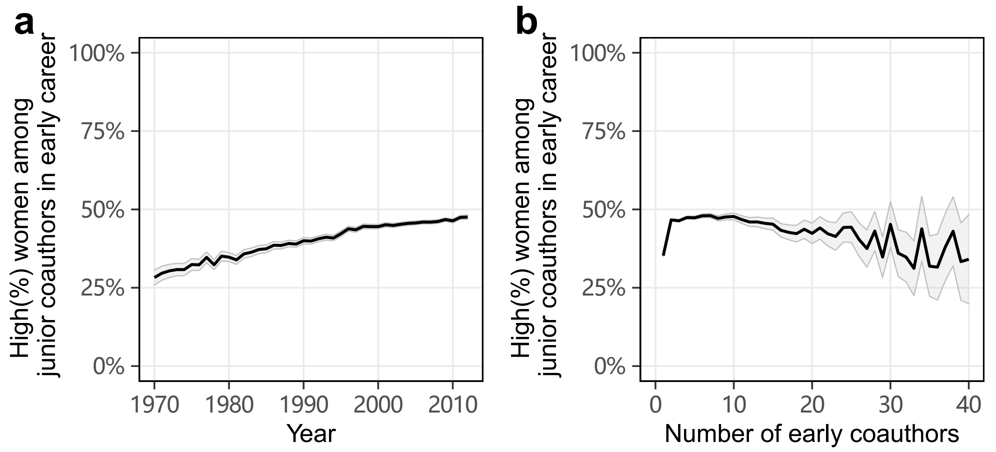

Supplementary Figure 11. **Descriptive statistics of the coauthorship network in the null model.** **a**, we show the proportion of researchers that have a high percentage of women junior coauthors in the early career over time. **b**, we show the proportion of researchers that have a high percentage of women junior coauthors in the early career as a function of the number of coauthors. Lines denote mean values and shaded areas represent 95% confidence intervals.

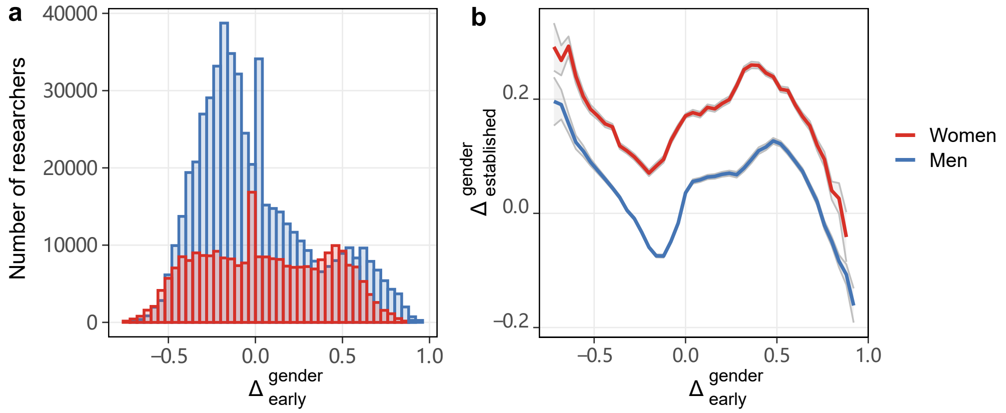

Supplementary Figure 12. **Gender diversity socialization in the early and established period.** We use $\Delta_{early}^{\text{gender}}$ to denote the difference between the gender diversity of a researcher's early coauthors relative to the null model. **a**, the number of researchers and early career gender diversity of coauthors. **b**, associating the gender diversity socialization in the early and established period of individual researchers. Lines denote mean values and shaded areas represent 95% confidence intervals in **b**.

for men researchers (Supplementary Figure 16**a**). These results suggest that the main findings of the gender diversity association effect are robust to the specific definition of the established period.

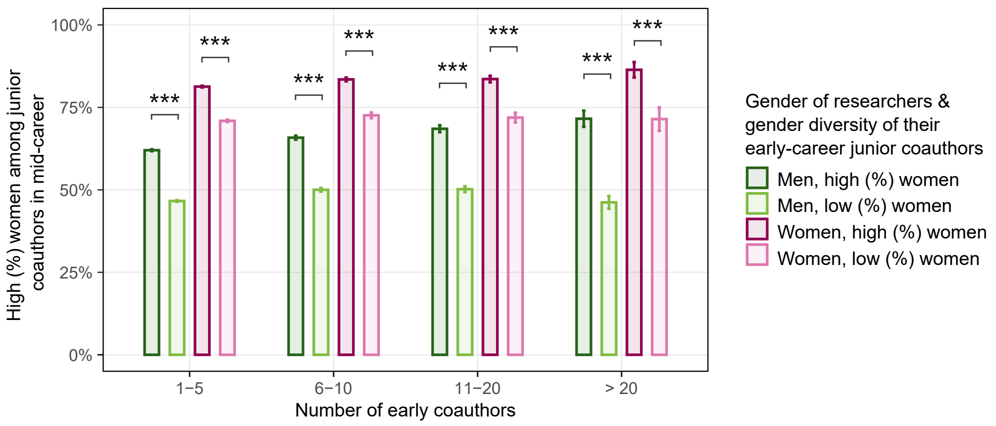

Supplementary Figure 13. **Gender diversity association effect in the coauthorship network relative to the null model as a function of the number of early coauthors.** We divide selected researchers by the number of their early coauthors into four groups ($n = 562,494$). Bars represent mean values and error bars indicate 95% confidence intervals. Two-sided t-tests are used for multiple comparisons. ($^{***}p < 0.001$; $^{**}p < 0.01$; $^{*}p < 0.05$).

We conduct another robustness test by incorporating two additional control variables into the null model, which is the first publishing year of authors and the career cumulative citation counts of senior authors. Citation counts are often used to assess the impact of researchers, yet this metric varies substantially over time and across fields. For example, citation counts received by a recently published paper can be times more than a paper published half a century ago. The number of citations a materials science paper garners is also substantially larger than a paper on pure mathematics. Since our standard of citation impact should be reasonable and applicable across several decades from 1970 to 2020, and for all subfields in arts and humanities, natural sciences, mathematics, engineering, and social sciences, we use an approximation of 100 citations as a lower bar of research impact and 1,000 citations as an upper level of impact for individual researchers. As such, we divide researchers into four tiers based on their total citation counts up to the year of the collaboration, with the first tier being authors receiving over 1,000 citations, the second tier being authors who have between 300 and 1,000 citations, the third tier being authors that have received less than 300 but more than 100 citations, and the rest being the fourth tier. In the new

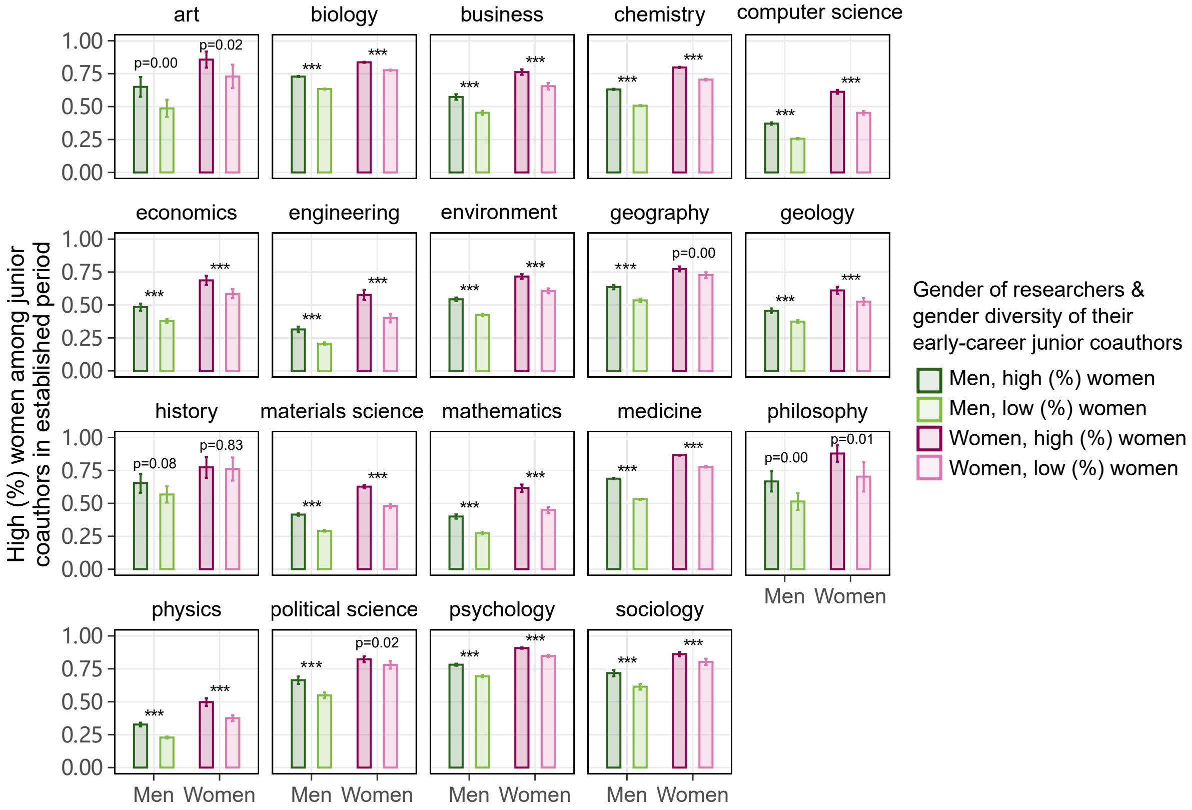

Supplementary Figure 14. **Gender diversity association effect in the coauthorship network relative to the null model by field.** We show how the diversity association effect varies by research fields in STEM, arts & humanities, and social sciences ($n = 562,494$). Bars represent mean values and error bars indicate 95% confidence intervals. Two-sided t-tests are used for multiple comparisons. ($^{***}p < 0.001$; $^{**}p < 0.01$; $^{*}p < 0.05$; NS, not significant).

null models, we find that the gender diversity association effect persists for established researchers in both the aggregate analysis and the time dynamics (Supplementary Figure 17).

Supplementary Note 3.6 Regression analyses

We conduct several regression analyses controlling for a series of factors that may affect the gender diversity association effects in the coauthorship network. Analogous to the regression analyses in the mentorship network, we incorporate several control variables including institutional prestige,

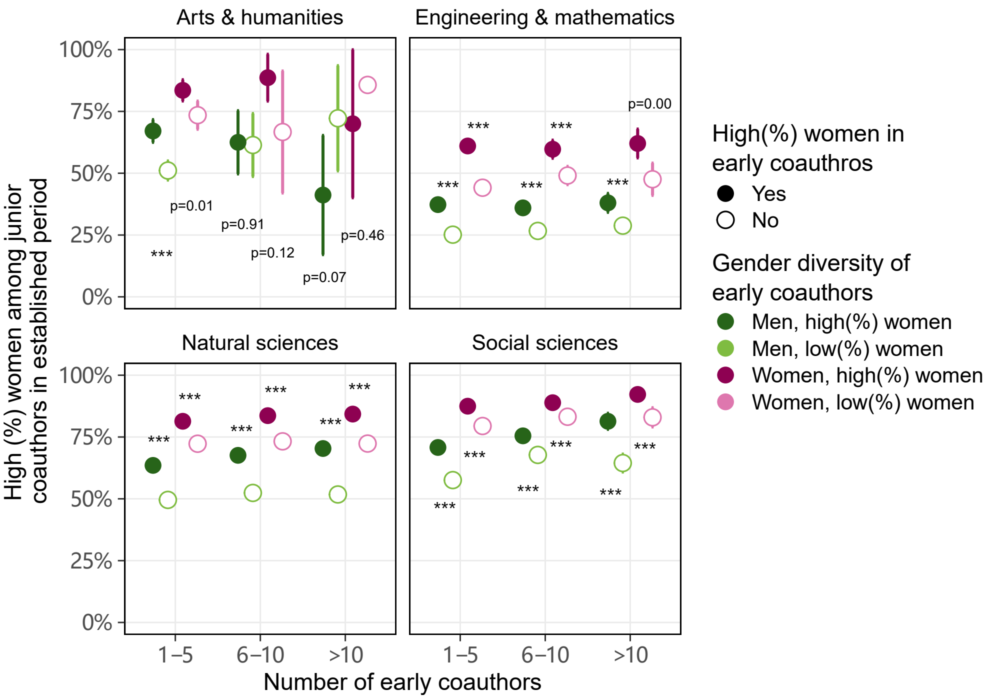

Supplementary Figure 15. **Gender diversity association effect in the coauthorship network by research domains and the number of early coauthors.** We show how the gender diversity association effect varies by research fields in four domains, i.e., arts & humanities, engineering & mathematics, natural sciences, and social sciences ($n = 562,494$). Dots denote mean values and error bars represent 95% confidence intervals. Two-sided t-tests are used for multiple comparisons. ($^{***}p < 0.001$; $^{**}p < 0.01$; $^{*}p < 0.05$).

the number of junior coauthors in the early career, and researcher gender. We also include two other variables of gender diversity to quantify the proportion of women junior coauthors of established researchers at the subfield and country levels.

The key variable to analyze is how the gender diversity of the coauthors during the early-career period affects the gender diversity of junior coauthors when researchers enter the established period. We first use linear regression models to examine how the women percentage among junior coauthors in the early career of researchers predicts the outcome variable, which is the women percentage of junior coauthors when they become established researchers (Supplementary Table 3).

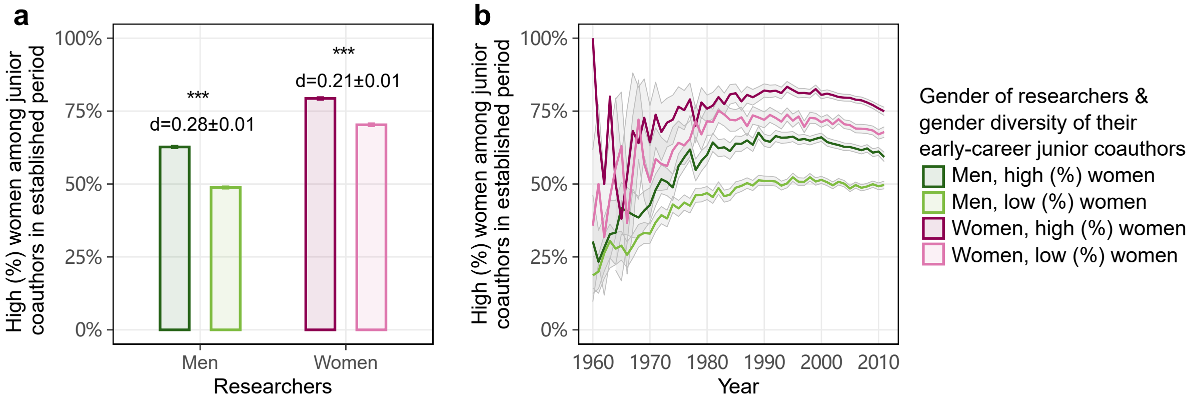

Supplementary Figure 16. **Robustness tests using 10 years as the established period.** In the main paper, we define the established period as the years after the 6th publishing career year of a researcher. Here we set the threshold of the established period to 10 years and repeat the analysis as a robustness test. **a**, the gender diversity association effect persists for both men and women established researchers ($n = 431,987$). **b**, the gender diversity association effect over time is similar to that in the main text. We use Cohen's d to indicate effect sizes. Bars represent mean values and error bars indicate 95% confidence intervals in **a**. Lines denote mean values and shaded areas represent 95% confidence intervals in **b**. Two-sided t-tests are used for multiple comparisons in **a**. ($^{***}p < 0.001$; $^{**}p < 0.01$; $^{*}p < 0.05$).

Several control variables, including researcher gender, the proportion of women junior researchers in the subfield, and the proportion of women junior researchers by country, all have strong positive relations with the women percentage among researchers' junior coauthors in the established period. Regarding the key variable of women percentage among early-career coauthors, we find that women percentage of coauthors in the early career has a significantly positive relation with the outcome variable, which is consistent across all linear regression models (Supplementary Table 3 models 10-12).

Under the null model setting, we then use logistic regression models to examine how having a high percentage of women coauthors in the early career of researchers predicts the outcome variable, which is a binary coding of whether the women percentage of junior coauthors is high relative to the null model when they become established researchers (Supplementary Table 4).

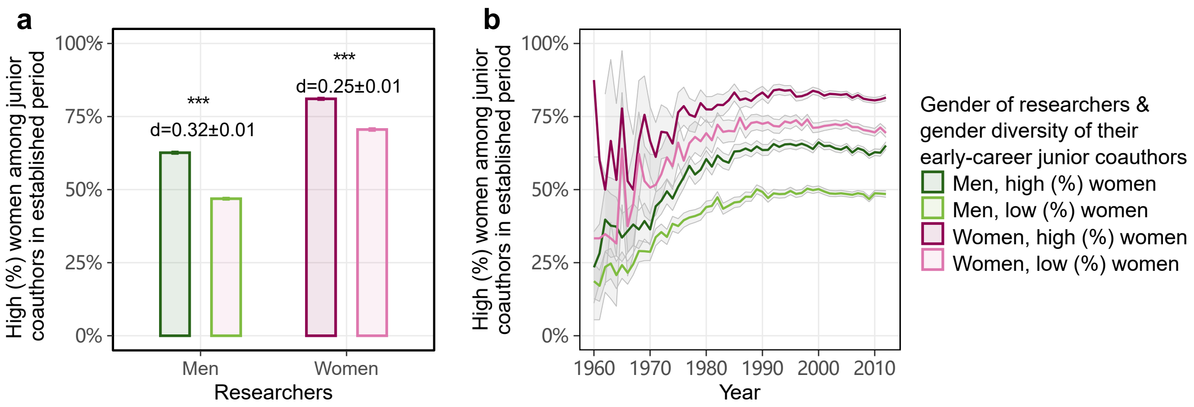

Supplementary Figure 17. **Robustness tests by inserting first publishing year and senior author citation counts in the null model.** We regard the established period as the years after the 6th publishing career year of a researcher. **a**, the gender diversity association effect persists for established researchers ($n = 562,494$). **b**, the gender diversity association effect over time is similar to that in the main text. We use Cohen's d to indicate effect sizes. Bars represent mean values and error bars indicate 95% confidence intervals in **a**. Lines denote mean values and shaded areas represent 95% confidence intervals in **b**. Two-sided t-tests are used for multiple comparisons in **a**. ($^{***}p < 0.001$; $^{**}p < 0.01$; $^{*}p < 0.05$).

Similarly, several control variables including researcher gender, the proportion of women junior coauthors in the subfield, and the proportion of women junior coauthors by country all have strong positive relations with having high women percentage among researchers' junior coauthors (Supplementary Table 4 model 16). For instance, when the focal researcher is woman, she has an odds ratio of 2.034, suggesting that she has on average 103.4% more likely to collaborate with a high percentage of women junior researchers compared to the null model. More importantly, we find that having high percentage of women among the early-career junior coauthors has a significantly positive relation with the outcome variable. The odds ratio of having a high percentage of women coauthors in the early-career is 1.495, suggesting that researchers who worked with a high percentage of early-career women coauthors are 49.5% more likely to collaborate with a cohort of gender diverse junior researchers when they become established researchers. The diversity socialization factor in the training period is consistently significant across all logistic regression models

(Supplementary Table 4 models 14-16).

These results suggest that, using either the crude proportion of women junior coauthors in the early career or the binary coding of whether the researcher worked with a high percentage of women junior coauthors relative to the null model, can validate the gender diversity association effect of how the gender diversity experience in the early career affects the diversity preferences of the researchers in the established period. The effects remain significant when we control for a number of potentially confounding factors, including time period, the number of junior coauthors, and other gender-related features such as the gender of the focal researcher, and the gender composition of junior researchers within a subfield and country.

Supplementary Table 3. **Linear regression models to predict the proportion of women among researchers' late junior coauthors.** Two-sided t-tests are used for multiple comparisons.

Dependent variable:	Women (%) coauthors in established period			
Model:	(9)	(10)	(11)	(12)
(Intercept)	0.386^{***}	0.343^{***}	0.028^{***}	-0.323^{***}
	(0.002)	(0.002)	(0.002)	(0.003)
Institutional prestige	0.032^{***}	0.028^{***}	-0.004^{**}	-0.005^{***}
	(0.002)	(0.002)	(0.002)	(0.001)
No. early junior coauthors	0.002^{***}	0.001^{***}	0.000	0.000^{***}
	(0.000)	(0.000)	(0.000)	(0.000)
Researcher is woman	0.160^{***}	0.131^{***}	0.103^{***}	0.091^{***}
	(0.001)	(0.001)	(0.001)	(0.001)
Women(%) by subfield			0.797^{***}	0.806^{***}
			(0.003)	(0.003)
Women(%) by country				0.754^{***}
				(0.005)
Women(%) in early coauthors		0.154^{***}	0.103^{***}	0.086^{***}
		(0.001)	(0.001)	(0.001)
R^2	0.081	0.128	0.214	0.243
Adj. R^2	0.081	0.128	0.214	0.243
Num. obs.	562253	562253	562253	562253

Robust standard-errors in parentheses

Signif. Codes: $^{***}p < 0.001$; $^{**}p < 0.01$; $^{*}p < 0.05$

Supplementary Table 4. **Logistic regression models to predict whether researchers have a high percentage of women junior coauthors (compared to the null model) in the established career.** Two-sided t-tests are used for multiple comparisons.

Dependent variable:	High (%) women coauthors in established period			
Model:	(13)	(14)	(15)	(16)
(Intercept)	-0.203^{***}	-0.417^{***}	-3.134^{***}	-6.082^{***}
	(0.013)	(0.013)	(0.019)	(0.031)
Institutional prestige	0.300^{***}	0.291^{***}	0.031^{*}	0.004
	(0.013)	(0.013)	(0.014)	(0.014)
No. early junior coauthors	0.016^{***}	0.013^{***}	0.002^{**}	0.005^{***}
	(0.001)	(0.001)	(0.001)	(0.001)
Researcher is woman	1.087^{***}	0.996^{***}	0.797^{***}	0.710^{***}
	(0.006)	(0.007)	(0.007)	(0.007)
Women(%) by subfield			6.635^{***}	6.826^{***}
			(0.032)	(0.032)
Women(%) by country				6.226^{***}
				(0.051)
High(%) women in early coauthors		0.628^{***}	0.490^{***}	0.402^{***}
		(0.006)	(0.006)	(0.006)
AIC	720525.740	708865.377	659025.448	643270.839
BIC	720570.699	708921.576	659092.887	643349.517
Log Likelihood	−360258.870	−354427.689	−329506.724	−321628.420
Deviance	720517.740	708855.377	659013.448	643256.839
Num. obs.	562253	562253	562253	562253

Robust standard-errors in parentheses

Signif. Codes: $^{***}p < 0.001$; $^{**}p < 0.01$; $^{*}p < 0.05$

Supplementary Note 4 Racial diversity socialization

Supplementary Note 4.1 Racial diversity socialization in coauthorship under the null model

The null model for the racial diversity socialization analysis in the coauthorship networks is analogous to the one we use for the gender diversity study (see Algorithm 2). The only difference is that after fully randomizing the coauthorship networks, instead of measuring the gender diversity, we compute the racial diversity level expected under the null model. The racial diversity for a researcher's coauthors is always defined as the value compared to the expected level of racial diversity under the null model setting. We provide descriptive statistics for the effects of racial diversity association in coauthorship networks. We show the number of established researchers selected for the analysis over time based on whether they have racially diverse early-career coauthors or not as a function of their first year in the training period (Supplementary Figure 18).

We use $\Delta^{\text{race}}_{\text{early}} = \rho_{\text{early}} - \langle \rho^{\text{null}}_{\text{early}} \rangle$ to denote the difference between the racial diversity of a researcher's early coauthors relative to the null model. We examine the diversity socialization effects $\Delta^{\text{race}}_{\text{established}}$ in the established period as a function of racial diversity $\Delta^{\text{race}}_{\text{early}}$ among early career coauthors. We find that researchers have an average $\Delta^{\text{race}}_{\text{early}} \simeq -0.2$, suggesting that researchers tend to have a cohort of early coauthors below the expected level of racial diversity (Supplementary Figure 19**a**). The racial diversity of coauthors in the established period $\Delta^{\text{race}}_{\text{established}}$ is monotonically increasing as a function of the racial diversity of early coauthor $\Delta^{\text{race}}_{\text{early}}$ (Supplementary Figure 19**b**).

To further explore the relation between the number of early coauthors and the racial diversity association effects, we break down the cohort of selected established researchers into four groups.

We find that the racial diversity association effect is larger for men researchers who worked with a larger number of early coauthors (Supplementary Figure 20**a**). More precisely, we show the racial diversity association of established researchers as a function of the number of their junior coauthors in the early career, and find the racial diversity association effect enhances as the number of early coauthors increases (Supplementary Figure 20**b**).

Different from gender diversity, racial diversity in the scientific workforce tends to have less variation across fields. We show the racial diversity association effect in the coauthorship network relative to the null model by field, particularly in STEM, arts & humanities, and social sciences (Supplementary Figure 21). In the majority of fields, researchers who worked with a racially diverse cohort of junior coauthors in the early career, relative to the null model, would have around 40% of chance to continue working with racially diverse junior coauthors in the established period. In comparison, researchers whose early coauthors are not racially diverse under the null model setting would have only about 20% probability of working with racially diverse coauthors when they become established researchers.

The racial/ethnic composition of the population varies substantially across countries, which also affects the racial diversity of the scientific workforce within a specific country. Therefore, the racial diversity association effect may be closely related to the country in which researchers reside. We select the top 20 countries with the largest number of established researchers, and show the proportion of researchers that have racially diverse junior coauthors in the early career relative to the null model (Supplementary Figure 23). In most countries, less than 30% of researchers worked with racially diverse coauthors in the early career. American countries appear to have the highest percentage of researchers working with racially diverse coauthors, while East Asian countries have the lowest probability of collaborating with racially diverse coauthors.

We further examine how the racial diversity association effect varies in four major domains,

i.e., arts & humanities, engineering & mathematics, natural sciences, and social sciences (Supplementary Figure 22). The racial diversity association effect is not significant in arts & humanities, which includes arts, history, and philosophy, especially for researchers with 10 or more early coauthors. In other domains, including engineering & mathematics, natural sciences, and social sciences, the racial diversity association effect is fairly consistent, suggesting that research fields may have a moderate influence on racial diversity socialization.

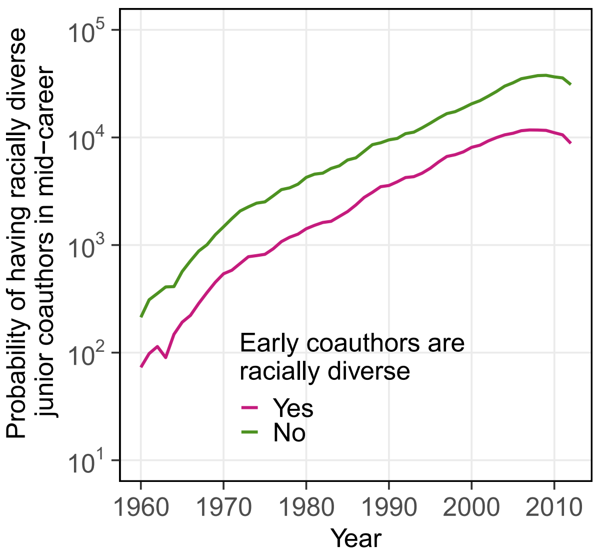

Supplementary Figure 18. **Descriptive statistics of the coauthorship network in the racial analysis.** We show the number of established researchers that have racially diverse junior coauthors in the early career relative to the null model, from 1960 to 2010.

Supplementary Note 4.2 Other robustness tests

We provide a robustness test using 10 years as the established period for the racial analysis, defined as the years after the 6th publishing career year of a researcher in the main paper. We find that the racial diversity association effect persists for established researchers, and the result is consistent for cohorts of researchers with different numbers of coauthors (Supplementary Figure 24**a**).

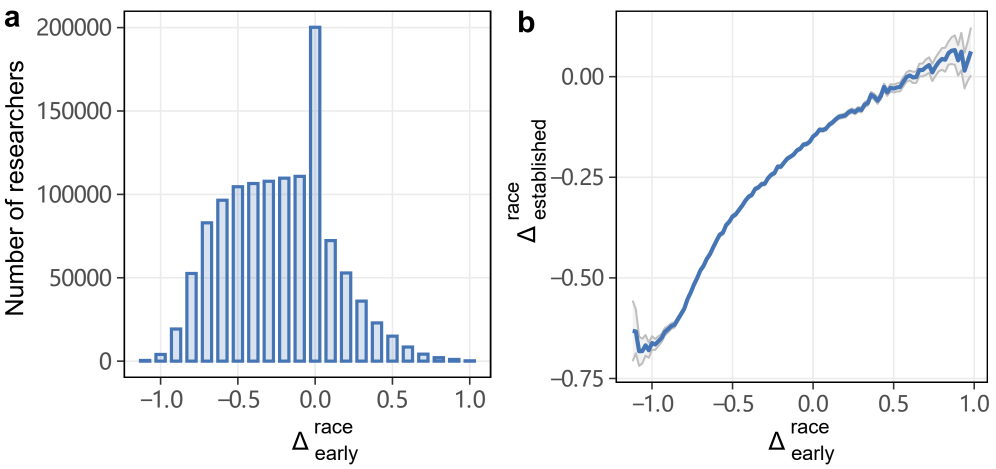

Supplementary Figure 19. **Racial diversity socialization in the early and established period.** We use $\Delta^{\text{race}}_{early}$ to denote the difference between the racial diversity of a researcher's early coauthors relative to the null model. **a**, the number of researchers and early career racial diversity of coauthors. **b**, associating the racial diversity socialization in the early and established period of individual researchers. Shaded areas represent 95% confidence intervals.

The racial diversity association effect over time is similar to the result reported in the main paper (Supplementary Figure 24**b**).

We conduct another robustness test by incorporating two additional control variables into the null model, which is the first publishing year of authors and the career cumulative citation counts of senior authors. After running the new null models, we find that the racial diversity association effect persists for established researchers in both the aggregate analysis and the time dynamics (Supplementary Figure 25).

Supplementary Note 4.3 Regression analyses

We conduct two regression analyses controlling for a series of factors that may affect the racial diversity association effects in the coauthorship network. Analogous to the regression analyses

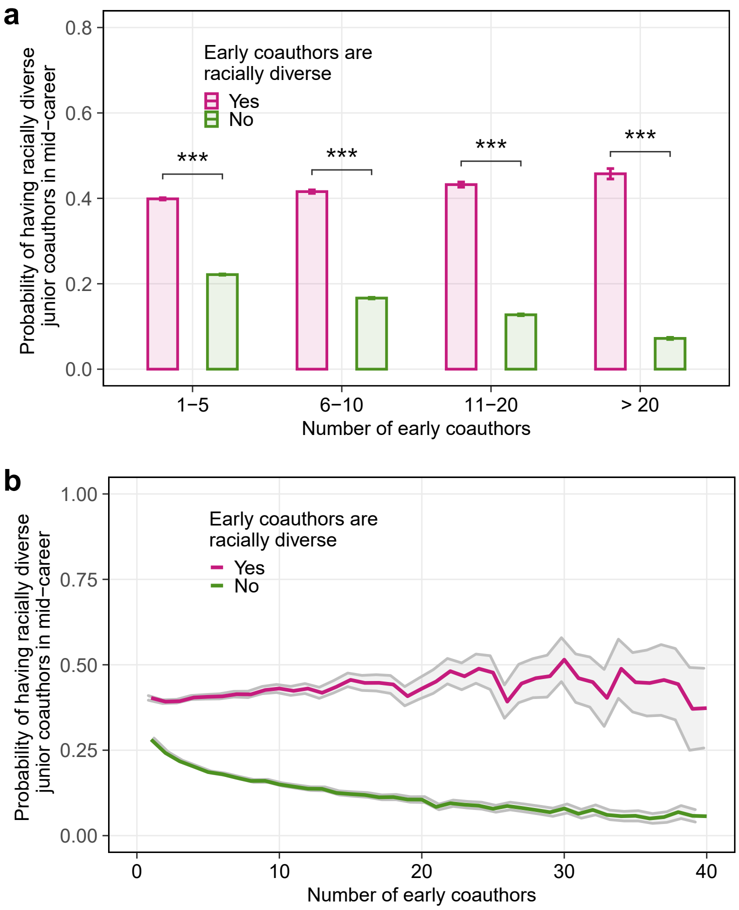

Supplementary Figure 20. **Racial diversity association as a function of the number of early coauthors. a**, we divide the established researchers in four groups according to the number of their junior coauthors in the early career ($n = 855,526$). **b**, we show the racial diversity association of established researchers as a function of the number of their junior coauthors in the early career. Bars represent mean values and error bars indicate 95% confidence intervals in **a**. Lines denote mean values and shaded areas represent 95% confidence intervals in **b**. Two-sided t-tests are used for multiple comparisons in **a**. ($^{***}p < 0.001$; $^{**}p < 0.01$; $^{*}p < 0.05$).

in the gender diversity analysis, we several control variables including institutional prestige, and the number of junior coauthors in the early career. We also include two other variables of racial diversity to quantify racial diversity at the subfield and country levels among junior coauthors of established researchers.

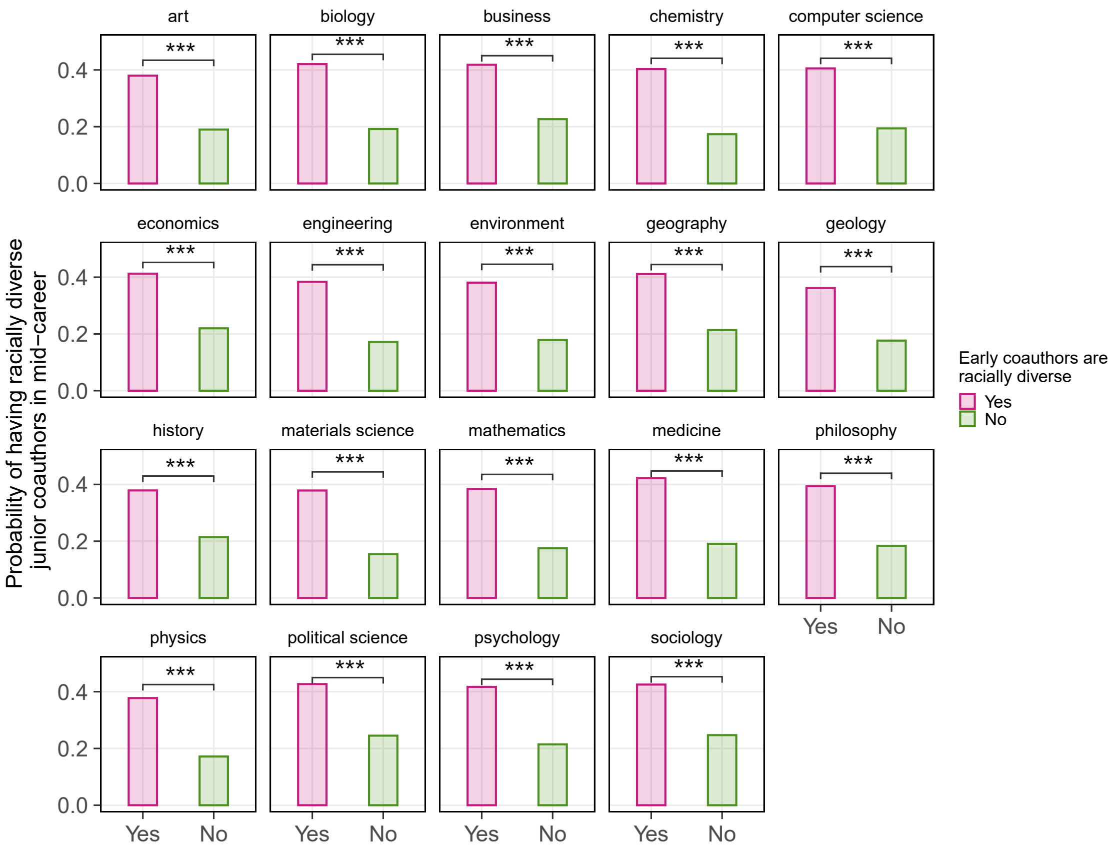

Supplementary Figure 21. **Racial diversity association effect in the coauthorship network relative to the null model by field.** We show how the racial diversity association effect varies by research fields in STEM, arts & humanities, and social sciences. Two-sided t-tests are used for multiple comparisons.($^{***}p < 0.001$; $^{**}p < 0.01$; $^{*}p < 0.05$).

The key variable to analyze is how the racial diversity of the junior coauthors during the early-career period affects the racial diversity of junior coauthors they collaborate with when researchers enter the established period. We first use linear regression models to examine how the racial diversity of junior coauthors in the early career of researchers predicts the outcome variable, which is the racial diversity of junior coauthors when they become established researchers (Supplementary Table 5). In the linear regression models, racial diversity is defined as the diversity score above the median value without using the null models. Control variables including the racial diversity of junior researchers in the subfield and the racial diversity of junior researchers by coun-

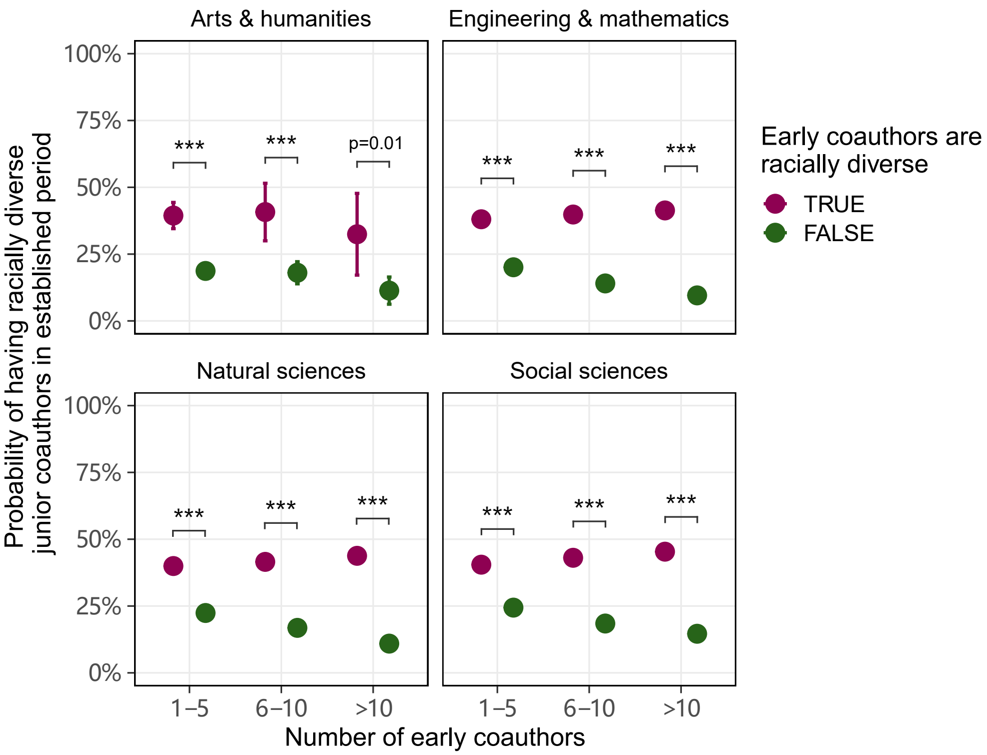

Supplementary Figure 22. **Racial diversity association effect in the coauthorship network by research domains and the number of early coauthors.** We show how the racial diversity association effect varies by research fields in four domains, i.e., arts & humanities, engineering & mathematics, natural sciences, and social sciences ($n = 855,526$). Dots represent mean values and error bars indicate 95% confidence intervals. Two-sided t-tests are used for multiple comparisons. ($^{***}p < 0.001$; $^{**}p < 0.01$; $^{*}p < 0.05$).

try have strong positive relations with the racial diversity of researchers' junior coauthors in the established period. Regarding the key variable of racial diversity of early-career coauthors, we find that the racial diversity of coauthors in the early career has a significantly positive relation with the outcome variable, which is consistent across all linear regression models (Supplementary Table 5 models 18-20).

Under the null model setting, we then use logistic regression models to examine how having racially diverse junior coauthors in the early career of researchers predicts the outcome variable, which is a binary coding of whether the racial diversity of junior coauthors is high relative to the

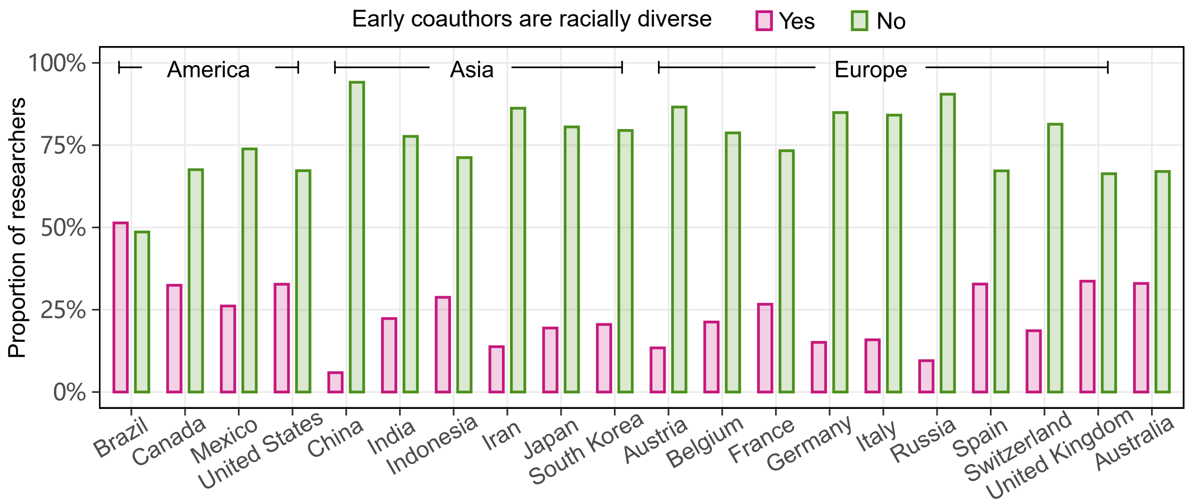

Supplementary Figure 23. **Proportion of researchers that have racially diverse junior coauthors in the early career.** We select the top 20 countries with the largest number of established researchers. We show the proportion of researchers that have racially diverse junior coauthors in the early career relative to the null model.

null model when they become established researchers (Supplementary Table 6). Similarly, control variables including the racial diversity of junior coauthors in the subfield, and the racial diversity of junior coauthors by country all have strong positive relations with having racially diverse junior coauthors in the established period (Supplementary Table 6 model 24). More importantly, we find that having racially diverse early-career junior coauthors has a significantly positive relation with the outcome variable. The odds ratio of having racially diverse junior coauthors in the early-career is 1.917, suggesting that researchers who worked with a racially diverse early-career junior coauthors are 91.7% more likely to collaborate with a cohort of racially diverse junior researchers when they become established researchers. The racial diversity socialization factor in the early career is consistently significant across all logistic regression models (Supplementary Table 6 models 22-24).

These results suggest that, using either the crude racial diversity of junior coauthors in the early career or the binary coding of whether the researcher worked with racially diverse junior

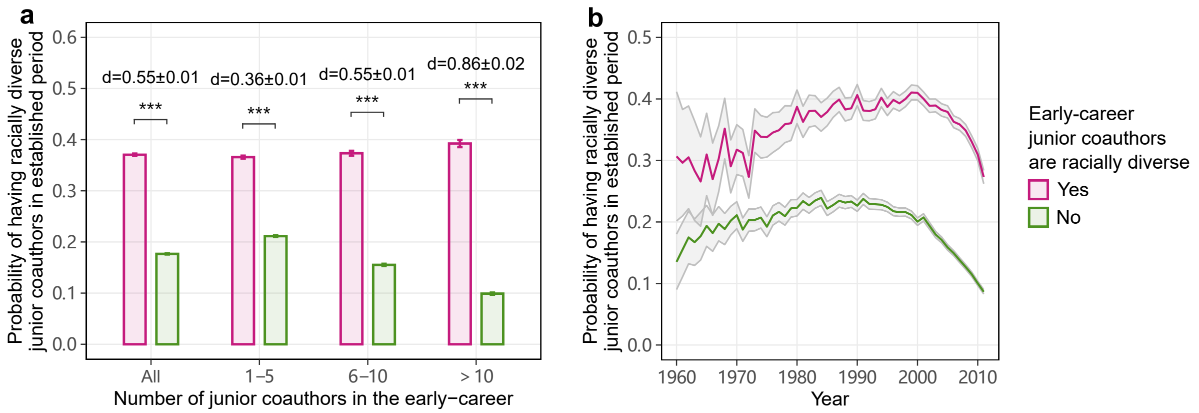

Supplementary Figure 24. **Robustness tests using 10 years as the established period for the racial analysis.** In the main paper, we define the established period as the years after the 6th publishing career year of a researcher. Here, we set the threshold of the established period to 10 years and repeat the racial diversity analysis as a robustness test. **a**, the racial diversity association effect persists for established researchers ($n = 664,850$). **b**, the racial diversity association effect over time is similar to that in the main text. We use Cohen's d to indicate effect sizes. Bars represent mean values and error bars indicate 95% confidence intervals in **a**. Lines denote mean values and shaded areas represent 95% confidence intervals in **b**. Two-sided t-tests are used for multiple comparisons in **a**. ($^{***}p < 0.001$; $^{**}p < 0.01$; $^{*}p < 0.05$).

coauthors relative to the null model, can validate the racial diversity association effect of how the racial diversity experience in the early career affects the diversity preferences of the researchers in the established period. The effects remain significant when we control for a number of potentially confounding factors, including time period, the number of junior coauthors, and other race-related features, such as the racial composition of junior researchers within a subfield and country.

Supplementary Table 5. **Linear regression models to predict the racial diversity among researchers' junior coauthors in the established period.** Two-sided t-tests are used for multiple comparisons.

Dependent variable:	Racial diversity of coauthors in established period			
Model:	(17)	(18)	(19)	(20)
(Intercept)	0.687^{***}	0.477^{***}	0.014^{***}	-0.127^{***}
	(0.002)	(0.002)	(0.004)	(0.003)
Institutional prestige	0.057^{***}	0.054^{***}	0.048^{***}	0.032^{***}
	(0.002)	(0.002)	(0.001)	(0.001)
No. early junior coauthors	-0.006^{***}	-0.007^{***}	-0.006^{***}	-0.001^{***}
	(0.000)	(0.000)	(0.000)	(0.000)
Racial diversity by field			0.692^{***}	0.181^{***}
			(0.006)	(0.005)
Racial diversity by country				0.880^{***}
				(0.002)
Racial diversity in early coauthors		0.404^{***}	0.388^{***}	0.123^{***}
		(0.001)	(0.001)	(0.001)
R^2	0.030	0.191	0.205	0.412
Adj. R^2	0.030	0.191	0.205	0.412
Num. obs.	853884	853884	853884	853884

Robust standard-errors in parentheses

Signif. Codes: $^{***}p < 0.001$; $^{**}p < 0.01$; $^{*}p < 0.05$

Supplementary Table 6. **Logistic regression models to predict whether researchers have high racial diversity among junior coauthors (compared to the null model) in the established career.** Two-sided t-tests are used for multiple comparisons.

Dependent variable:	High racial diversity of coauthors in established period			
Model:	(21)	(22)	(23)	(24)
(Intercept)	-1.185^{***}	-1.582^{***}	-3.630^{***}	-5.908^{***}
	(0.013)	(0.013)	(0.036)	(0.040)
Institutional prestige	0.339^{***}	0.355^{***}	0.319^{***}	0.131^{***}
	(0.013)	(0.013)	(0.013)	(0.014)
No. early junior coauthors	-0.040^{***}	-0.033^{***}	-0.033^{***}	-0.014^{***}
	(0.000)	(0.000)	(0.000)	(0.000)
Racial diversity by field			3.022^{***}	0.482^{***}
			(0.049)	(0.052)
Racial diversity by country				5.611^{***}
				(0.023)
High racial diversity in early coauthors		1.084^{***}	1.066^{***}	0.651^{***}
		(0.005)	(0.006)	(0.006)
AIC	926460.633	888271.608	884358.118	800880.839
BIC	926495.606	888318.238	884416.405	800950.784
Log Likelihood	−463227.317	−444131.804	−442174.059	−400434.420
Deviance	926454.633	888263.608	884348.118	800868.839
Num. obs.	853884	853884	853884	853884

Robust standard-errors in parentheses

Signif. Codes: $^{***}p < 0.001$; $^{**}p < 0.01$; $^{*}p < 0.05$

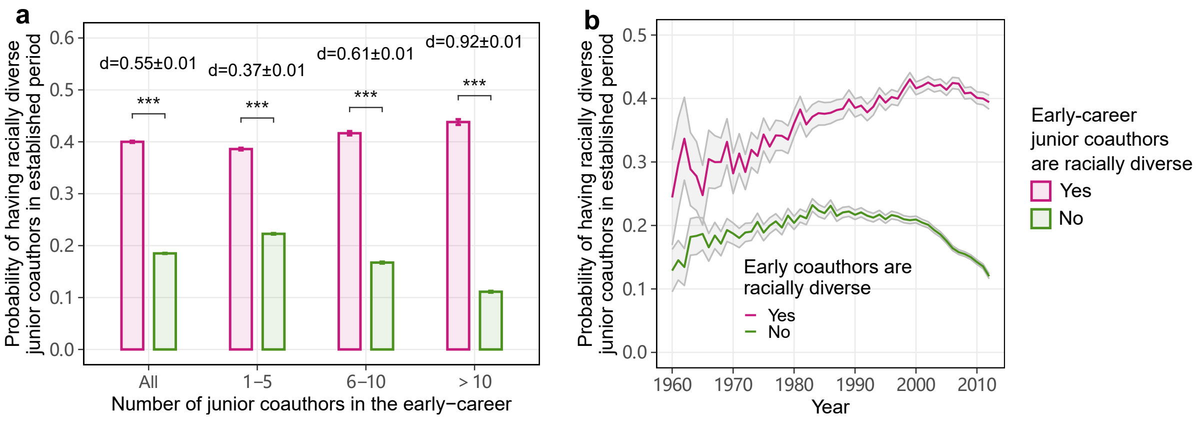

Supplementary Figure 25. **Robustness tests by inserting first publishing year and senior author citation counts in the null model.** We regard the established period as the years after the 6th publishing career year of a researcher. **a**, the racial diversity association effect persists for established researchers ($n = 855,526$). **b**, the racial diversity association effect over time is similar to that in the main text. We use Cohen's d to indicate effect sizes. Bars represent mean values and error bars indicate 95% confidence intervals in **a**. Lines denote mean values and shaded areas represent 95% confidence intervals in **b**. Two-sided t-tests are used for multiple comparisons in **a**. ($^{***}p < 0.001$; $^{**}p < 0.01$; $^{*}p < 0.05$).

Supplementary Note 5 Sociological theory and implications

For sociodemographic categories related to gender and race/ethnicity, which are our focus here, we should expect the diversity association effect to be stronger for White men due to their tendency to be overrepresented in most academic fields, compared to women or members of other racial groups. Researchers who received training in relatively gender diverse research groups are substantially more likely to advise women advisees when they become established researchers. This effect is particularly strong for men who were trained in large research groups. In collaboration networks, established researchers tend to have woman-majority junior coauthors if their own early-career social network was composed of woman-majority junior coauthors. Similarly, we find that established researchers are substantially more likely to collaborate with more racially/ethnically diverse junior coauthors if they worked with racially/ethnically diverse junior coauthors during

their early-career period.

We investigate the extent to which early-career scientific collaborators with representationally diverse cohorts of scholars are associated with forming or participating in more gender/racially diverse research groups when they become established researchers. We call this tendency the diversity association effect, which suggests that how established researchers build diverse research groups is closely related to the representational diversity of groups in their training period. In order to measure such diversity association effects, we defined a null model that randomizes the mixing patterns of social networks, preserving each individual's number of interactions, while controlling for covariates that may affect homophily and the formation of social ties, including time, subfield, country, and institutional prestige. We then measure the magnitude of diversity association effects as the difference between observed diversity and the expected diversity, under the null model. In this way, we define diversity at the group level, relative to the population as a whole, rather than relative to any particular individual, and a researcher can be a part of a diverse group even if they have majority social identity.

Applied to two large, longitudinal data sets, we find that being trained in an early-career environment with a relatively high proportion of gender or racial diversity is strongly associated with a researcher going on to collaborate with diverse junior collaborators as an established researcher themselves. This effect appears consistently in both mentorship and coauthorship networks, and large research groups produce stronger diversity association effects for early-career researchers, as they naturally provide more opportunities for cross-gender/race interactions.

Homophily is a ubiquitous pattern in the distribution of social relationships in society and academia. For instance, within the scientific community, men are more likely to mentor and collaborate with other men and women are more likely to mentor and collaborate with other women, than would be expected based on random mixing alone. For members of under-represented social

groups, homophily can provide tangible benefits for academic careers, including identity-specific social support and increasing the likelihood of persisting in the academic pipeline[19,20]. However, our results suggest that researchers trained in gender or racially diverse environments are more likely to diversify their social connections and lead diverse environments when they become established researchers. That is, experience with sociodemographically diverse environments during the formative years of the early career tends to somehow enable or facilitate researchers in a way that can mitigate the extant tendency toward homophily in academic social relationships.

The long careers of established researchers further imply that these diversity association effects, whether large or small, are likely to play out for many years, and similarly, the lack of experience with diversity in the past continues to have an effect on the gender and racial composition of research groups today. Even in non-academic scenarios, recent research has found that childhood exposure to Black peers will impact the residential choices of White people when they become adults, and increase the chances that they live in neighborhoods with more Black people[21]. The suggested mechanism is a change in white people's preferences, which is consistent with our results.

The impact of these early-career experiences can be thought of as a form of post-functionalist socialization[22,23]—specifically, a process we call "diversity socialization"—in which training in a representationally diverse social environment facilitates a researcher's own construction of behaviors, attitudes, beliefs, or preferences for how they will later construct and lead a research group. The particular mechanism might operate through the development of preferences for such collaborations, or through developing a form of cultural capital that helps an established researcher recruit representationally or be attractive to diverse early-career scholars. Selection effects may also play a role, as joining a particular research group with a particular established researcher or particular demographic composition is both a structured and socially negotiated process.

We observe a strong diversity association effect in both gender and race in scientific collaboration networks. The external factors that drive these dynamics may have different socio-economic mechanisms. Gender diversity has strong fluctuation by fields, and in particular, social science fields like psychology and sociology exhibit stronger gender diversity association effects (Supplementary Figure 8). On the other hand, the effect is weaker in arts & humanities fields, especially history. Although some STEM fields, such as materials science and physics, have relatively higher racial diversity association effects than social science fields like political science, there appears to be less variance in terms of racial diversity association effects across fields (Supplementary Figure 21).

Gender and racial diversity are strongly influenced by the demographics of each particular country. The name-based gender/race labeling approach we employed may also have an impact on some effect sizes. The majority of researchers in all major racial groups can be assigned a racial distribution based on last names, while most Asian researchers cannot obtain accurate gender labeling with first names. This may give the racial analysis a more complete mapping of diversity socialization processes in academia.

Large-scale longitudinal data on racial homophily and doctoral mentorship in small-group settings would be particularly valuable; the data set used here is too limited in size to provide clear estimates of this form of sociodemographic diversity[1], and hence our estimates are mainly derived from medium- and large-sized research groups.

Supplementary Note 6 Intersectional analysis of gender and race

Intersectional effects of gender and race have been studied in the sociology of science[2], and we examine here in this section whether there is any intersectional diversity socialization effect in science. We use intersectional effects of both gender and race in the early career to predict gender diversity of junior coauthors in the established period, and find that racial diversity in the early career has little predictive power for gender diversity in the established period (Supplementary Figure 26**a**). Similarly, gender diversity in the early career has little predictive power for racial diversity in the established period (Supplementary Figure 26**b**).

We present logistic regression models to test intersectional diversity socialization effects. As in the previous sections, we use post-2000, institutional prestige, and the number of junior coauthors in the early career as control variables. We also include gender and racial diversity by subfield and country to control for variations in other aspects of the diversity socialization process.

We test whether racial diversity in the early career predicts the gender diversity of junior coauthors in the established period of researchers. The early-career gender diversity factor and variables controlling for gender diversity at the subfield and country level have consistent and positively significant relation with the gender diversity in the established period (Supplementary Table 7). However, we find that early-career racial diversity has no stable predictability of gender diversity (Supplementary Table 7 models 26-28). Other racial diversity factors, including those controlling the racial diversity at the level of subfield and country, have no clear pattern in predicting the gender diversity association effect.

Similarly, although the early-career racial diversity factor and variables controlling for racial diversity at the subfield and country level have consistent and positively significant relations with

the racial diversity in the established period, we find that early-career gender diversity has no stable predictability of racial diversity (Supplementary Table 8). Other gender diversity factors, including those controlling gender diversity at the level of subfield and country, have no clear pattern in predicting racial diversity association effects. These results suggest that the diversity socialization process is specific to the exact diversity environment that researchers experience in the early career, and intersectional diversity association effects appear to be non-significant.

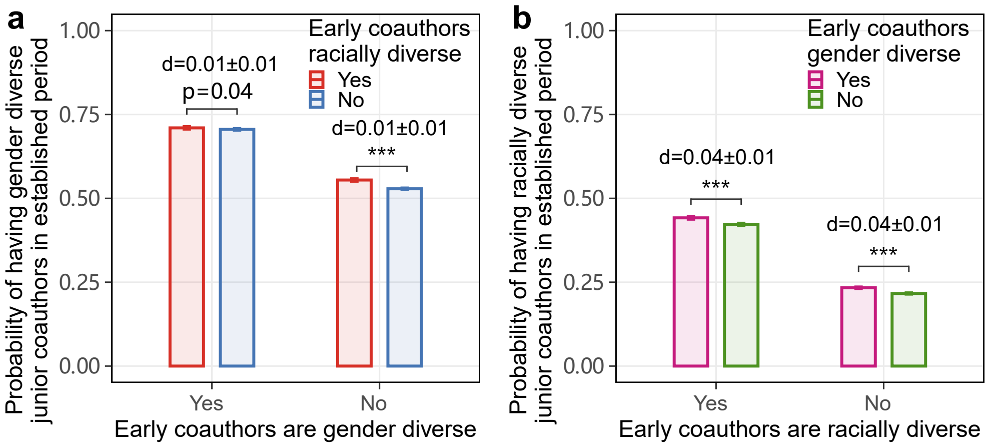

Supplementary Figure 26. **Intersectional effects of gender and race in the early career to predict diversity socialization in the established period. a**, intersectional effects of both gender and race in the early career to predict gender diversity of junior coauthors in the established period ($n = 440,941$). **b**, intersectional effects of both gender and race in the early career to predict racial diversity of junior coauthors in the established period ($n = 440,941$). We use Cohen's d to indicate effect sizes. Bars represent mean values and error bars indicate 95% confidence intervals. Two-sided t-tests are used for multiple comparisons. $^{***}p < 0.001$; $^{**}p < 0.01$; $^{*}p < 0.05$).

Supplementary Table 7. **Logistic regression models to predict whether researchers have high gender diversity among junior coauthors (compared to the null model) in the established career.** Two-sided t-tests are used for multiple comparisons.

Dependent variable:	High gender diversity of coauthors in established period			
Model:	(25)	(26)	(27)	(28)
(Intercept)	-8.157^{***}	-7.996^{***}	-2.761^{***}	-4.905^{***}
	(0.069)	(0.069)	(0.078)	(0.082)
Post-2000	0.149^{***}	0.130^{***}	0.196^{***}	0.165^{***}
	(0.007)	(0.007)	(0.007)	(0.007)
Institutional prestige	0.002^{***}	0.002^{***}	0.001^{***}	0.001^{***}
	(0.000)	(0.000)	(0.000)	(0.000)
No. early junior coauthors	0.003^{***}	0.003^{***}	0.001	0.003^{***}
	(0.001)	(0.001)	(0.001)	(0.001)
Racial diversity by subfield	13.680^{***}	13.114^{***}	0.518^{***}	0.938^{***}
	(0.116)	(0.117)	(0.145)	(0.147)
Racial diversity by country	0.175^{***}	0.115^{***}	-0.131^{***}	-0.597^{***}
	(0.024)	(0.026)	(0.026)	(0.028)
Researcher is woman	0.986^{***}	0.909^{***}	0.765^{***}	0.674^{***}
	(0.008)	(0.008)	(0.008)	(0.008)
Women(%) by subfield			6.326^{***}	6.487^{***}
			(0.043)	(0.044)
Women(%) by country				5.904^{***}
				(0.056)
High racial diversity in early coauthors		0.017^{*}	0.032^{***}	-0.006
		(0.008)	(0.008)	(0.008)
High(%) women in early coauthors		0.569^{***}	0.489^{***}	0.394^{***}
		(0.007)	(0.007)	(0.007)
AIC	549284.882	541987.982	517804.980	505944.181
BIC	549361.859	542086.952	517914.946	506065.144
Log Likelihood	-274635.441	-270984.991	-258892.490	-252961.091
Deviance	549270.882	541969.982	517784.980	505922.181
Num. obs.	440941	440941	440941	440941

Robust standard-errors in parentheses

Signif. Codes: $^{***}p < 0.001$; $^{**}p < 0.01$; $^{*}p < 0.05$

Supplementary Table 8. **Logistic regression models to predict whether researchers have high racial diversity among junior coauthors (compared to the null model) in the established career.** Two-sided t-tests are used for multiple comparisons.

Dependent variable:	High racial diversity of coauthors in established period			
Model:	(29)	(30)	(31)	(32)
(Intercept)	-2.984^{***}	-3.117^{***}	-4.775^{***}	-7.043^{***}
	(0.030)	(0.031)	(0.080)	(0.084)
Post 2000	-0.038^{***}	-0.073^{***}	-0.073^{***}	0.021^{**}
	(0.007)	(0.007)	(0.007)	(0.007)
Institutional prestige	0.005^{***}	0.005^{***}	0.005^{***}	0.004^{***}
	(0.000)	(0.000)	(0.000)	(0.000)
No. early junior coauthors	-0.016^{***}	-0.011^{***}	-0.013^{***}	-0.014^{***}
	(0.001)	(0.001)	(0.001)	(0.001)
Women(%) by subfield	0.572^{***}	0.474^{***}	-0.038	-0.489^{***}
	(0.033)	(0.034)	(0.041)	(0.042)
Women(%) by country	3.920^{***}	3.571^{***}	3.589^{***}	1.862^{***}
	(0.055)	(0.057)	(0.057)	(0.065)
Researcher is woman	0.001	-0.004	-0.003	0.009
	(0.007)	(0.008)	(0.008)	(0.008)
Racial diversity by subfield			3.200^{***}	3.127^{***}
			(0.143)	(0.146)
Racial diversity by country				5.302^{***}
				(0.036)
High(%) women in early coauthors		0.011	0.010	0.021^{**}
		(0.007)	(0.007)	(0.007)
High racial diversity in early coauthors		0.932^{***}	0.928^{***}	0.635^{***}
		(0.007)	(0.007)	(0.008)
AIC	518451.687	501751.426	501250.617	473287.124
BIC	518528.663	501850.396	501360.584	473408.087
Log Likelihood	-259218.843	-250866.713	-250615.309	-236632.562
Deviance	518437.687	501733.426	501230.617	473265.124
Num. obs.	440941	440941	440941	440941

Robust standard-errors in parentheses

Signif. Codes: $^{***}p < 0.001$; $^{**}p < 0.01$; $^{*}p < 0.05$

Supplementary References

1. Ke, Q., Liang, L., Ding, Y., David, S. V. & Acuna, D. E. A dataset of mentorship in bioscience with semantic and demographic estimations. *Sci. Data* **9**, 1–12 (2022).

2. Kozlowski, D., Larivière, V., Sugimoto, C. R. & Monroe-White, T. Intersectional inequalities in science. *Proc. Natl. Acad. Sci. USA* **119**, e2113067119 (2022).

3. Lockhart, J. W., King, M. M. & Munsch, C. L. Name-based demographic inference and the unequal distribution of misrecognition. *Nat. Hum. Behav.* (2023).

4. Wang, K., Shen, Z., Huang, C., Wu, C.-H., Dong, Y. & Kanakia, A. Microsoft academic graph: When experts are not enough. *Quant. Sci. Stud.* **1**, 396–413 (2020).

5. Lockhart, J. W., King, M. M. & Munsch, C. Name-based demographic inference and the unequal distribution of misrecognition. *Nat. Hum. Behav.* 1–12 (2023).

6. Huang, J., Gates, A. J., Sinatra, R. & Barabási, A.-L. Historical comparison of gender inequality in scientific careers across countries and disciplines. *Proc. Natl. Acad. Sci. USA* **117**, 4609–4616 (2020).

7. Wapman, K. H., Zhang, S., Clauset, A. & Larremore, D. B. Quantifying hierarchy and dynamics in US faculty hiring and retention. *Nature* **610**, 120–127 (2022).

8. Leslie, S.-J., Cimpian, A., Meyer, M. & Freeland, E. Expectations of brilliance underlie gender distributions across academic disciplines. *Science* **347**, 262–265 (2015).

9. Coate, K. & Howson, C. K. Indicators of esteem: gender and prestige in academic work. *Brit. J. Sociol. Educ.* **37**, 567–585 (2016).

10. Santos, J. M., Horta, H. & Amâncio, L. Research agendas of female and male academics: a new perspective on gender disparities in academia. *Gender Educ.* **33**, 625–643 (2021).

11. Jones, B. F., Wuchty, S. & Uzzi, B. Multi-university research teams: Shifting impact, geography, and stratification in science. *Science* **322**, 1259–1262 (2008).

12. Wuchty, S., Jones, B. F. & Uzzi, B. The increasing dominance of teams in production of knowledge. *Science* **316**, 1036–1039 (2007).

13. Yang, Y., Tian, T. Y., Woodruff, T. K., Jones, B. F. & Uzzi, B. Gender-diverse teams produce more novel and higher-impact scientific ideas. *Proc. Natl. Acad. Sci. USA* **119**, e2200841119 (2022).

14. Wu, L., Wang, D. & Evans, J. A. Large teams develop and small teams disrupt science and technology. *Nature* **566**, 378–382 (2019).

15. Cheryan, S., Ziegler, S. A., Montoya, A. K. & Jiang, L. Why are some STEM fields more gender balanced than others? *Psychol. Bull.* **143**, 1 (2017).

16. Stoet, G. & Geary, D. C. The gender-equality paradox in science, technology, engineering, and mathematics education. *Psychol. Sci.* **29**, 581–593 (2018).

17. Jayachandran, S. The roots of gender inequality in developing countries. *Annu. Rev. Econ.* **7**, 63–88 (2015).

18. US Census Bureau, Frequently occurring surnames from the 2010 Census. `https://www.census.gov/topics/population/genealogy/data/2010_surnames.html` (2010). Accessed: 2022-09-30.

19. Hofstra, B., McFarland, D. A., Smith, S. & Jurgens, D. Diversifying the professoriate. *Socius* **8** (2022). URL `https://api.semanticscholar.org/CorpusID:247727155`.

20. McGaskey, F. G., Freeman, S., Guyton, C., Richmond, D. D. & Guyton, C. W. The social support networks of black males in higher education administration doctoral programs:

An exploratory study. *West. J. Black Stud.* **40**, 141–158 (2016). URL `https://api.semanticscholar.org/CorpusID:54705549`.

21. Merlino, L. P., Steinhardt, M. F. & Wren-Lewis, L. The long run impact of childhood interracial contact on residential segregation. *J. Public Econ.* **239**, 105242 (2024).

22. Xie, Y. & Shauman, K. A. Sex differences in research productivity: New evidence about an old puzzle. *Am. Sociol. Rev.* 847–870 (1998).

23. Guhin, J., Calarco, J. M. & Miller-Idriss, C. Whatever happened to socialization? *Annu. Rev. Sociol.* **47**, 109–129 (2021).
